# The Progress and Outlook of Multivalent‐Ion‐Based Electrochromism

**DOI:** 10.1002/smsc.202300025

**Published:** 2023-09-17

**Authors:** Bing Xu, Jingwei Chen, Ziyi Ding, Jianwei Hu, Yingxin Zhang, Haizeng Li, Huanlei Wang

**Affiliations:** ^1^ School of Materials Science and Engineering Ocean University of China Qingdao 266100 China; ^2^ Institute of Frontier & Interdisciplinary Science Shandong University Qingdao 266237 China

**Keywords:** coordination, electrochromism, electrodeposition, intercalation, multivalent

## Abstract

Electrochromic technology has witnessed numerous achievements in recent years both in research and commercialization. Electrochromic devices (ECD) based on different electrochemical mechanism have been developed for various applications, ranging from smart windows, thermal management, rear views, display, camouflage, etc. Compared to conventional ECDs based on monovalent charge carriers (e.g., H^+^, Li^+^), incorporating multivalent ions with rich electrochemistry, high charge density, and small ionic radius has opened new possibilities in novel ECDs. The merits of multivalent ions are harvested in ECDs activated by ion intercalation/deintercalation (e.g., Zn^2+^, Al^3+^, Ca^2+^, Mg^2+^), reversible metal electrodeposition (e.g., Cu^2+^, Bi^3+^, Zn^2+^), and dynamic metal–ligand interactions (e.g., Cu^2+^, Fe^2+^). Herein, the working mechanism, characteristics, and up‐to‐date achievements in multivalent ECDs are summarized and classified accordingly. The applications of multivalent ECDs for smart windows, energy storage, thermal management, multicolor displays, etc., are exemplified. The issues and challenges encountered by multivalent ECDs are emphasized, and the future directions for developing multivalent ECDs are also summarized. The aim of this review is to inspire more efforts in the exploration and the proliferation of multivalent ECDs.

## Graphical abstract

Multivalent metal ions are endowed with rich electrochemistry and have shown promise in various electrochemical devices. Great achievements in multivalent‐metal‐ions‐based electrochromic devices (ECDs), for example, have been witnessed in recent years. The working principle, issues, advances, applications, and future perspectives in multivalent‐metal‐ions‐based ECD are summarized in this review, aiming at accelerating the commercialization of multivalent ECDs.

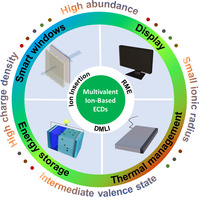

## 1 Introduction

**Figure 1:**
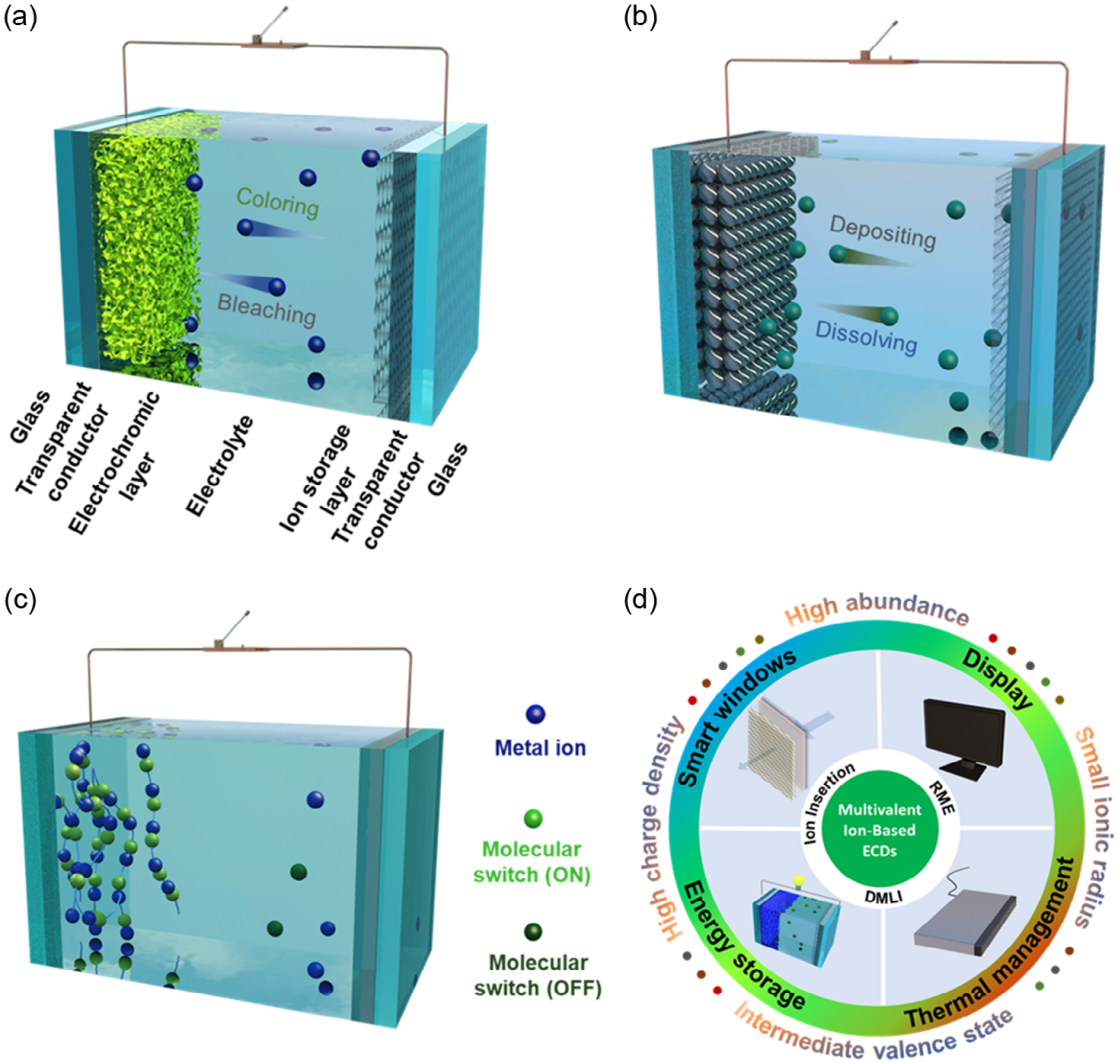
a–c) Configuration and working principle of electrochromic devices (ECD) based on ion intercalation/deintercalation (a), reversible metal electrodeposition (RME) (b), and dynamic metal‐ligand interaction (DMLI) (c). d) The application of multivalent‐ion‐based ECDs.

Electrochromism refers to the optical properties (transmittance, absorbance, and reflectance) variation in an electrochromic material (ECM) or electrochromic device (ECD), upon application of electric pulses (current or potential).^[^ ^1^ ^]^ Due to the reversible optical and electrochemical behavior, low energy consumption, and charge‐storage capability, ECDs have attracted extensive attention in various fields, especially in smart windows,^[^ ^2^ ^]^ displays,^[^ ^3^ ^]^ and optically adjustable electronic components.^[^ ^4^, ^5^ ^]^ A conventional ECD is a sandwich‐like structure consisting of transparent substrate, transparent conductor, electrochromic layer, electrolyte, and ion‐storage layer (**Figure** **1**a).^[^ ^6^ ^]^ Among them, the electrochromic layer is the key component of reversible optical modulation. As schemed in Figure 1a, the basic working principle of a conventional ECD is ion‐intercalation/deintercalation‐induced electrochromism. Under appropriate voltage or current modulation, ECMs are reduced and colored (for cathodic coloration materials), while reversed electric bias can oxidize and bleach the ECMs.^[^ ^1^ ^]^ The whole electrochemical and electrochromic process is reversible. The performances of ECDs are not only dependent on the electrochemical reactions of heterogeneous electron transfer between electrodes and the electrochromic (EC) layer/ion‐storage layer, but also on the homogeneous ion transfer through the EC layer, the ion‐transport layer and the ion‐storage layer.^[^ ^7^ ^]^

In the past few decades, due to the limited choices of high‐performance ECMs, it is challenging for conventional ECDs to harvest large optical contrast (Δ*T*), fast switching, long durability, and color diversity, thus hindering the development of EC technology.^[^ ^8^ ^]^ Therefore, researchers have been committed to developing novel ECMs to improve the performance of ECDs. Strategies including doping, introducing pore structures, and defect engineering of ECMs have been adopted to improve the performance of ECDs.^[^ ^9^ ^]^ However, it is still difficult and challenging to obtain superior ECDs only by structure engineering of ECMs.^[^ ^10^ ^]^ Introduction of alternative electrochemistry is promising in designing novel ECDs. Aside from conventional ion‐intercalation/deintercalation‐induced electrochromism, ECDs based on other electrochemical mechanism are also being developed, including reversible metal electrodeposition (RME) and dynamic metal–ligand interaction (DMLI).^[^ ^11^, ^12^ ^]^ RME‐based ECDs rely on the reversible electroplating/stripping of a metallic layer onto/from the working electrode.^[^ ^13^ ^]^ Despite the different electrochemical mechanism between RME and traditional ECMs, considering that the reversible electroplating/stripping of metal layers are also accompanied by variations in optical properties, multivalent‐ion‐based RME is also incorporated in this review. The configuration of a typical RME–ECD is shown in Figure 1b, which is composed of a working electrode (e.g., indium tin oxide [ITO]) and a counter electrode (metal frame/grid or ion‐storage materials), sandwiching the electrolyte (containing dissolved metal ions). Upon cathodic scan, metal ions dissolved in the electrolyte will be electrodeposited onto the working electrode, forming a thin and dense metallic layer that reduces the transmittance (or increase the reflectance) of the ECDs. Anodic scan can initiate the oxidation of metallic layer into soluble metal ions, restoring the initial transparency of ECDs. RME‐based ECDs have become a promising substitute for traditional intercalation‐based ECDs,^[^ ^14^ ^]^ with advantages of fast switching speed, long‐term durability, neutral color, deep color state, uniformity, and so on.^[^ ^15^ ^]^ DMLI‐induced electrochromism is different from that of metallo–organic materials, for example, polypyridyl complexes.^[^ ^16^, ^17^ ^]^ Such metal complexes usually exhibit highly reversible redox reactions on metal centers. For example, when the metal center is in a low‐oxidation state, the low acceptor orbital charge‐transfer transition from metal to ligand may occur, accompanied by a strong color change.^[^ ^18^ ^]^ In contrast, DMLI‐induced electrochromism is based on the coordination/dissociation of metal ions with switchable dyes. The configuration of a typical DMLI–ECD is shown in Figure 1c, which is composed of two electrodes (e.g., ITO glass) sandwiching electrolytes of valence changeable metal ions and switchable dyes. Upon anodic scan, metal ions will be oxidized and coordinated with the dye, rendering colored states of the ECD. Reversibly, the metal ions will be cathodically reduced and dissociate with the dyes, restoring the initial transparency of the ECD. DMLI‐based ECDs generally show excellent coloration efficiency, redox stability, and color diversity.^[^ ^19^ ^]^

As for conventional intercalation‐based ECDs, monovalent ions with small radius and high charge density are favorable due to the ease in ion insertion/extraction into/from the host lattice.^[^ ^20^ ^]^ However, problems such as the strong corrosivity of H^+^, the high cost of Li, and the large radius of Na^+^ and K^+^ have inspired exploration of multivalent ions (e.g., Zn^2+^, Al^3+^, Mg^2+^, Ca^2+^, etc.)‐based ECDs.^[^ ^21^, ^22^ ^]^ Multivalent ions have attracted extensive attention in EC field due to their high abundance, low cost, high charge density, and small ionic radius.^[^ ^21^ ^]^ Compared with monovalent ions, multivalent‐ion‐based ECDs support multi‐electron transfer, and the presence of intermediate valence state can sometimes introduce extra color states in ECDs.^[^ ^13^, ^23^ ^]^ It has been demonstrated that multivalent ions have enabled construction of intercalation‐based ECDs with fast response time, large Δ*T* and long cycle stability,^[^ ^24^ ^]^ assembly of RME‐based ECDs with good electrochemical reversibility, intermediate colored state and long cycling stability,^[^ ^12^, ^23^ ^]^ and DMLI–ECDs with high coloration efficiency, color tunability, and high white light contrast ratio.^[^ ^11^ ^]^

The intriguing merits of multivalent metal ions, for example, enriched electrochemistry, high bulk energy density, and presence of intermediate oxidation states have brought advances into intercalation‐based ECDs and are also being employed in constructing superior RME‐based ECDs and DMLI‐based ECDs. There have been several excellent reviews summarizing the achievements in EC fields, including smart windows,^[^ ^1^ ^]^ metal–oxide nanocrystal‐based ECDs,^[^ ^25^ ^]^ Zn‐based ECDs,^[^ ^26^ ^]^ Al‐based ECDs,^[^ ^27^ ^]^ ECMs for displays,^[^ ^3^ ^]^ etc. Here in this review, we systematically describe the configuration, working mechanism, and characteristics of different types of ECDs and focus on summarizing and discussing the up‐to‐date developments in various type ECDs (intercalation‐, RME‐, and DMLI‐based) enabled by multivalent metal ions, for their applications in smart windows, energy storage, display, and thermal management (Figure 1d). The issues and challenges presiding in multivalent‐ion‐based ECDs are discussed, future prospects for the development of advanced multivalent‐ion‐based ECDs are also predicted. This review serves as a guideline to systematically introduce different types of multivalent ECDs, summarize the developments, and showcase their applications, aiming at inspiring further efforts and paving the way for the proliferation of multivalent‐ion‐based ECDs.

## 2 Discussion

ECDs based on ion intercalation/de‐intercalation, RME, and DMLIs have been fabricated, with adoption of multivalent metal ions. In this section, multivalent‐ion‐based ECDs will be discussed based on the electrochemical mechanism.

### 2.1 Ion‐Insertion‐Based Multivalent Electrochromism

ECMs based on ion insertion and extraction have attracted wide interest in both academic research and industry due to their attractive characteristics for commercial applications.^[^ ^28^ ^]^ Monovalent ions have various limitations, such as the corrosive nature of H^+^, high cost of Li, and large radius of Na^+^. In recent years, inspired by the low cost, small ionic radius, and high charge density, multivalent‐ions‐based electrochemical devices with higher‐charge‐storage capability have been developed, including batteries, hybrid capacitors, and ECDs.^[^ ^29^, ^30^, ^31^ ^]^ The number of publications related to multivalent‐ion‐intercalation‐based ECDs has increased rapidly in recent years. Multivalent‐ion‐ECDs based on ion (e.g., Zn^2+^, Al^3+^, Ca^2+^, Mg^2+^, etc.) intercalation/deintercalation will be discussed as follows.

#### 2.1.1 Zn^2+^‐Insertion‐Based Multivalent Electrochromism

**Figure 2:**
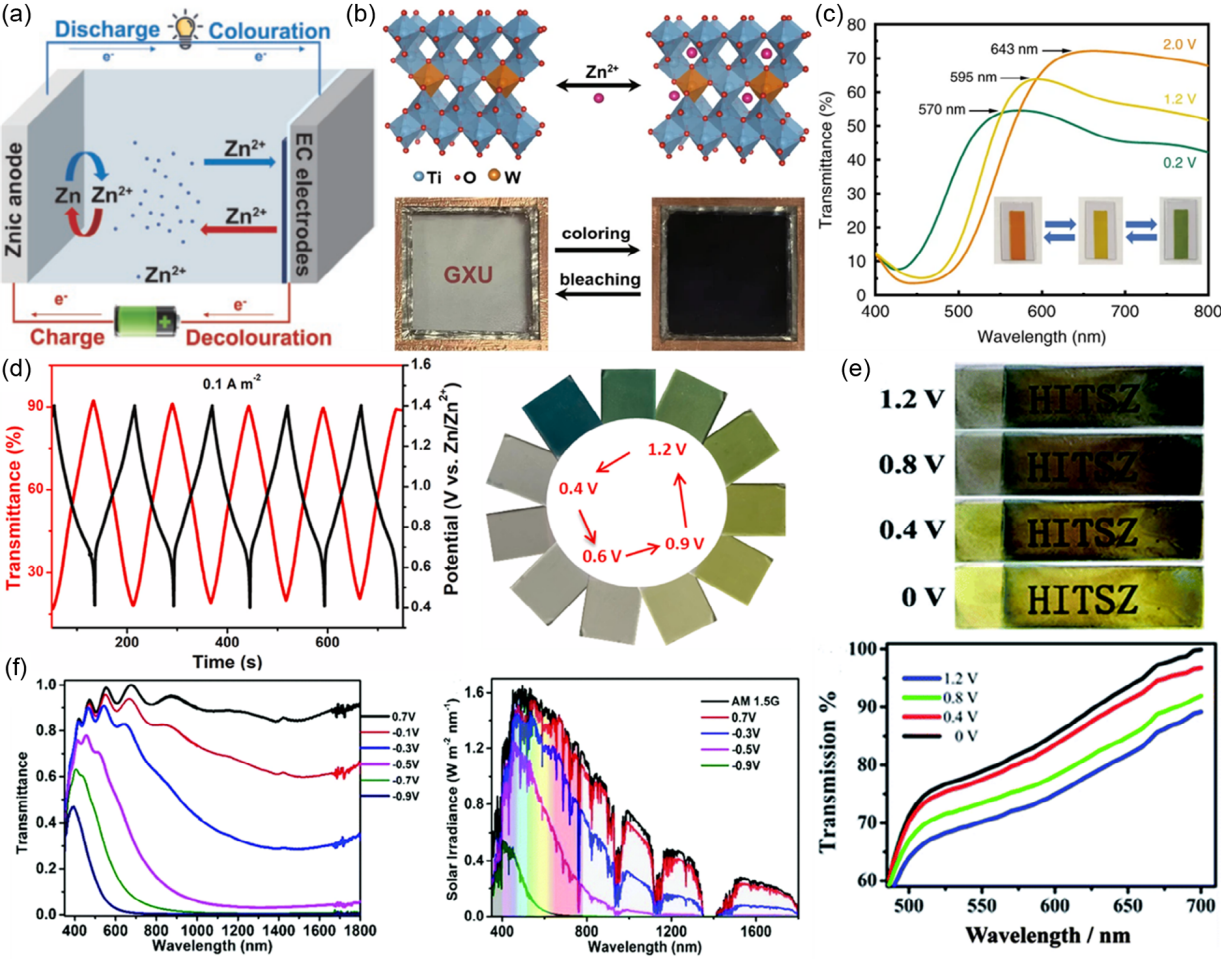
Demonstration of ion‐intercalation‐based multivalent ECDs. a) Schematic of a typical zinc‐ion‐based ECD. Reproduced with permission.^[^ ^36^ ^]^ Copyright 2022, Wiley‐VCH. b) Zinc‐ion intercalation into W–TiO_2_ and the photographs of W–TiO_2_–Zn ECD at transparent and colored states. Reproduced with permission.^[^ ^37^ ^]^ Copyright 2021, Springer Nature. c) Transmittance spectra of sodium‐ion‐intercalated VO_*x*_ (SVO) at different voltages, insets are the corresponding photographs. Reproduced under the terms of the CC‐BY Creative Commons Attribution 4.0 International license (https://creativecommons.org/licenses/by/4.0).^[^ ^39^ ^]^ Copyright 2020, The Authors, published by Springer Nature. d) Transmittance and potential profiles of polyaniline (PANI)–Zn ECD, and the photographs of the ECD at different potentials. Reproduced with permission.^[^ ^33^ ^]^ Copyright 2022, Elsevier. e) Photographs and transmittance spectra of polypyrrole (Ppy)–Zn ECD. Reproduced with permission.^[^ ^57^ ^]^ Copyright 2018, Royal Society of Chemistry. f) Transmittance spectra and solar irradiance spectra of m‐WO_3−*x*_ nanowires (NWs) films in Al^3+^ electrolyte. Reproduced with permission.^[^ ^5^ ^]^ Copyright 2018, Royal Society of Chemistry.

Zinc‐ion‐based electrochemical devices, for example, zinc‐ion batteries have attracted numerous attention due to their low cost, low redox potential, high theoretical specific capacity (820 mAh g^−1^), rich reserves of zinc, stability in water,^[^ ^32^ ^]^ and multivalent characteristics of zinc, which manifest higher charge density for a facile charge‐transfer process.^[^ ^33^ ^]^ In contrast, metal foil anodes, for example, Zn, suffer from dendrite formation, corrosion, and side reactions in aqueous electrolytes.^[^ ^34^ ^]^ At present, many studies have shown that these problems can be effectively alleviated by means of electrolyte engineering and electrode structure optimization.^[^ ^35^ ^]^ As a result, zinc‐ion‐intercalation‐based ECMs and ECDs have been explored in recent years. As shown in **Figure** **2**a, a typical zinc‐ion‐based multivalent ECD is composed of Zn anode, electrochromic electrode (cathode), and Zn^2+^ electrolyte.^[^ ^36^ ^]^ The electrochromism in Zn‐ECD can be initiated by intercalation/deintercalation into/from the ECMs upon discharge/charge. Several inorganic and organic materials have been investigated for zinc‐ion‐based electrochromism, including TiO_2_,^[^ ^37^, ^38^ ^]^ WO_3_,^[^ ^24^ ^]^ manganese oxide,^[^ ^24^ ^]^ vanadium oxide,^[^ ^39^, ^40^ ^]^ Prussian blue (PB),^[^ ^41^ ^]^ polyaniline (PANI),^[^ ^33^, ^42^ ^]^ etc.

Titanium dioxide, as a typical intercalation‐type metal oxide, has a variety of crystal forms, which shows excellent physical and chemical stability and acid resistance.^[^ ^43^ ^]^ However, it is found out that the strong lattice interaction between Zn^2+^ and anatase TiO_2_ frameworks hinder the diffusion kinetics, resulting in low capacity. In addition, rutile TiO_2_ has high thermal stability with a layered structure, shortened ion‐diffusion distance and more exposed active sites.^[^ ^38^ ^]^ Nonetheless, rutile TiO_2_ suffers from limited optical modulation, low coloration efficiency, and a relatively high intercalation energy.^[^ ^44^ ^]^ Nanostructured rutile TiO_2_ has been applied for Zn^2+^‐induced electrochromism, yet with a contrast of only 45% (@980 nm) and a limited coloration efficiency of only 13.6 cm^2^ C^−1^.^[^ ^38^ ^]^ The electrochromic performance of TiO_2_ nanocrystals are also evaluated in electrolytes with different valence metal ions (Li^+^, Zn^2+^, and Al^3+^). The superior electrochromic performance of TiO_2_ nanocrystals (larger Δ*T* and improved cycling stability) in Zn^2+^ electrolyte was ascribed to the lower potential barriers of Zn^2+^ (0.55 eV) in TiO_2_ compared to that of Li^+^ (0.59 eV) and Al^3+^ (0.74 eV), showcasing the superiority of multivalent‐ion‐based ECD than monovalent‐ion‐based ECDs.^[^ ^45^ ^]^ Nonetheless, it has been proved that the intercalation energy of ions can be reduced by doping.^[^ ^37^, ^46^ ^]^ W‐doped TiO_2_ has reduced Zn^2+^‐intercalation energy to 0.86 eV, enabling improved electrochemical performance in W–TiO_2_. The W–TiO_2_ shows improved Zn^2+^‐diffusion coefficient (8.46 × 10^−13^ cm^2^ s^−1^), large Δ*T* (66% @550 nm), fast switching speed (coloration time *τ*_c_ 9 s, bleaching time *τ*_b_ 2.7 s), high coloration efficiency (37.3 cm^2 ^C^−1^), and good cycling stability (8.2% Δ*T* loss after 1000 cycles) without phase change.^[^ ^37^ ^]^ As shown in Figure 2b, upon intercalation, the W–TiO_2_–Zn ECD can reach a deep blue color.

Tungsten oxide (WO_3_) is a typical and promising electrochromic metal oxide, which can reversibly switch between transparent and blue state, with high Δ*T* and good cycle stability in monovalent‐cations‐based electrolytes.^[^ ^47^, ^48^ ^]^ The electrochromic properties of WO_3_ in Zn^2+^ electrolyte can be improved through morphology control, and it has been found out that WO_3_ nanorods manifest faster switching kinetics (*τ*_c_/*τ*_b_ = 3 s/2.2 s), larger Δ*T* (72.4% @550 nm), and higher coloration efficiency (67.6 cm^2^ C^−1^) than WO_3_ nanoflakes, due to the higher surface area of nanorods.^[^ ^49^ ^]^ However, the diffusion of divalent ions in WO_3_ is hindered and the obtained capacity is limited. Li et al. exchanged W^6+^/Mo^4+^ and Ti^4+^ through liquid‐phase doping to obtain titanium‐substituted tungsten–molybdenum oxide (MTWO), leading to reduced particle size of MTWO, enhanced electrolyte accessibility, and shortened ion‐diffusion length. Given the similar radii of W^6+^, Mo^4+^ and Ti^4+^, monoclinic MTWO retained the perovskite‐like structure with abundant cationic vacancies, thus providing rigid channels and embedding sites for Zn^2+^, realizing improved capacity and electrochromic performance, and exhibiting an areal capacity of 260 mAh m^−2^ and high Δ*T* of 76%.^[^ ^24^ ^]^

The aforementioned multivalent‐ion‐intercalation‐based electrochromic inorganic TiO_2_ and WO_3_ normally have only two color states (transparent and colored), without multicolor tunability. Electrochromic vanadium‐based oxide, in contrast, shows multicolor characteristics due to the multivalency of *V*.^[^ ^50^ ^]^ Albeit large interlayer spacing, the limited electrical conductivity and hindered electrochemical kinetics have limited the electrochemical performance of VO_*x*_. Nanostructure engineering and doping are effective in enhancing the electrochemical performance of VO_*x*_. For example, solution‐processed colloidal vanadium oxide nanoparticles (NPs) manifest improved electrochemical kinetics,^[^ ^40^ ^]^ yet the cyclic stability is to be further optimized. Sodium‐ion‐intercalated VO_*x*_ (SVO) shows significantly improved electrochromic performance for Zn^2+^ intercalation, delivering green, yellow, and orange colors under different potential (Figure 2c).^[^ ^39^ ^]^ Fabry–Pérot (F–P) resonance is an alternative way to obtain multicolor by selectively tuning light reflection, which has been adopted to harvest multicolor in WO_3_^[^ ^51^ ^]^ and Mn_2_O_3_,^[^ ^24^ ^]^ etc. Manganese oxide is an excellent electrode for zinc‐ion batteries due to the 1D diffusion channel, high theoretical capacity,^[^ ^24^ ^]^ low cost, and high safety,^[^ ^52^ ^]^ yet with poor electrochromic performance and low color‐rendering efficiency. Employing the concept of F–P resonance, multicolor MnO_*x*_‐based ECD was fabricated. Through structure engineering, photonic metamaterials based on Mn_2_O_3_ top layer and Ti bottom layer were prepared. Seven bright colors can be obtained by adjusting the thickness of Mn_2_O_3_. The oxidation state of Mn can be adjusted by Zn^2+^ intercalation under different potentials, rendering a wider spectrum of colors. The Mn_2_O_3_–Zn‐based multicolor ECD also shows energy‐storage capability with a high capacity of 283 mAh g^−1^ at 0.2 A g^−1^.^[^ ^24^ ^]^

The option of multicolors have inspired the pursuit of 2D tunability in Commission internationale de l'éclairage (CIE) coordinates, instead of a straight line or a curve.^[^ ^36^ ^]^ Superposition of two electrochromic electrodes/devices, in this way, can achieve multicolor tunability covering a 2D region in CIE coordinates. For example, by overlaying two SVO electrodes sandwiching a piece of zinc foil in between, the ECD device can realize six color states (orange, amber, yellow, brown, chartreuse, and green), instead of only three colors for the SVO electrode.^[^ ^39^ ^]^ F–P resonance‐based multicolor materials are also adopted for superposition. With similar device configuration, SVO can be coupled with W/WO_3_ electrodes or WO_3_ electrode. When W/WO_3_ and SVO are superimposed, reflective multicolor ECDs can be obtained, while transmissive multicolor ECDs are obtained when superimposing WO_3_ and SVO. The combination of transmission and reflection multicolor ECDs can be used as electronic labels or light filters.^[^ ^36^ ^]^

Aside from the previously discussed inorganic metal oxides, organic materials are also of interest for Zn^2+^ electrochromic applications. PANI shows conjugated structure, good electronic conductivity, facile preparation, mechanical flexibility,^[^ ^53^ ^]^ fast switching speed, high optical contrast,^[^ ^54^ ^]^ and different oxidation states, and thus different colors under different potentials.^[^ ^33^ ^]^ However, the electrochemical cycling stability of PANI is limited, due to the fracture of the of PANI molecules skeleton and formation of soluble low‐molecular‐weight products. Nonetheless, nanostructure design and incorporation of multivalent ions and organic electrolytes can mitigate the polymer chain destruction and improve cycle stability. It was found out that the Δ*T* of PANI in aqueous and organic electrolyte was similar, but the switching kinetics (*τ*_c_/*τ*_b_ = 2.0/2.4 s), cycle stability (Δ*T* retention of 92.7% after 10 000 cycles) and coloration efficiency (211 cm^2^ C^−1^) were greatly improved in Zn(ClO_4_)_2_/PC electrolyte, due to the capacitive–dominant behavior, lower interfacial activation energy, higher conductivity and ion mobility, and better wettability of organic electrolyte.^[^ ^33^ ^]^ As shown in Figure 2d, PANI manifest electrochromic‐energy‐storage capability as well as multicolor tunability. Self‐doped PANI (SDPANI) was also synthesized to assemble Zn‐ECD. With a porous nylon@Au substrate, SDPANI and Zn were electrodeposited at two sides, fabricating a flexible energy‐storage ECD with multicolor tunability under different potentials.^[^ ^55^ ^]^ At the same time, PANI is also a dual‐band electrochromic polymer material. The so‐called dual‐band electrochromism refers to the ability to independently control the transmittance of two bands, such as visible (VIS) light and near‐infrared (NIR).^[^ ^41^, ^56^ ^]^ Electrodeposited PANI has both multicolor conversion ability (Figure 2d) and dual‐band electrochromic function. PANI can be converted to reduced leucoemeraldine base, semi‐oxidized emeraldine salt (ES), and fully oxidized pernigraniline salt (PS) under different applied potentials, modulating the VIS and NIR transmittance.^[^ ^42^ ^]^ By assembling two layers of PANI/ITO electrodes, a Zn frame counter electrode in between and 1 m Zn(ClO_4_)_2_/PC electrolyte, the resulting device offers three optical states: bright, cold, and dark. “Bright” refers to the higher transmittance of VIS and NIR, “dark” refers to the lower transmittance of both, while “cold” refers to the case when VIS transmittance is higher and NIR transmittance is lower. Therefore, controlling the oxidation state of PANI and the proportion of LES, ES, and PS through progressive electrochemical reaction is the key to realize the independent adjustment of NIR and VIS transmittance.^[^ ^42^ ^]^ Another conductive polymer, polypyrrole (Ppy) was also matched with Zn for assembly of ECD. Ppy and zinc were electrodeposited onto ITO‐coated poly(ethylene terephthalate) (PET) substrates, harvesting flexibility, and energy‐storage capability in the Zn–ECD. As shown in Figure 2e, the as‐assembled Zn–ECD shows different transmittance spectra under different potentials, and a black–yellow color transition.^[^ ^57^ ^]^ Another example of flexible Zn–ECD was obtained by adopting electrodeposited PANI composited with MnO_2_ and poly(3,4‐ethylenedioxythiophene) (PEDOT) polystyrene sulfonate on Ni@Ag nanowires (NWs)‐coated PET as cathode, assembling a flexible Zn–ECD with multicolor tunability and energy‐storage functions.^[^ ^58^ ^]^

It should be noted that in these reported Zn^2+^‐induced electrochromism, zinc foils are often employed to construct ECDs. However, the transparency of Zn foil is a concern, and pasting Zn foil at the side will cause blooming effect.^[^ ^59^ ^]^ Adoption of transparent zinc mesh, in contrast, is a viable option to avoid such issues.^[^ ^41^ ^]^

#### 2.1.2 Al^3+^‐Based Multivalent Electrochromism

Among multivalent‐ion‐based ECDs, Al‐based ECDs (AECDs) have attracted wide attention because of the smaller ionic radius (0.53 Å) and the three‐electron charge transfer of Al^3+^, which provides the possibility to create multifunctional novel devices with excellent electrochromic and energy‐storage performance.^[^ ^5^, ^60^ ^]^ However, research in AECDs is at an early stage and many problems exist for practical application. For example, the high charge density of Al^3+^, strong electrostatic interaction between the host material and Al^3+^, and formation of continuous and dense passivation layer on the surface of Al anode may lead to poor reaction kinetics, limited reversibility, and low cycle stability.^[^ ^61^ ^]^ Gong et al. assembled a multicolor Al^3+^‐electrochromic‐energy‐storage device composed of PANI cathode, aluminum frame anode and AlCl_3_ electrolyte.^[^ ^62^ ^]^ The device showed a discharge capacity of 68.1 mAh m^−2^, excellent rate capability, large Δ*T* (55%@630 nm), and high coloration efficiency (93.62 cm^2 ^C^−1^). To understand the intercalation/deintercalation mechanism of AECDs, Zhang et al. designed a Al^3+^‐based dual‐band electrochromic smart window with m‐WO_3−*x*_ NW as cathode, rendering “bright,” “cool,” and “dark” modes as shown in Figure 2f.^[^ ^5^ ^]^ The m‐WO_3−*x*_ NW film is completely transparent to both VIS and NIR at the 0.7 V “bright” mode. In the “cool” mode (−0.5 V), the film could block 92.6% of the solar heat in the NIR region, while maintaining a high VIS light transmittance (VLT) of 58.6%, which could significantly reduce the energy consumption of air conditioning and lighting in buildings. In the “dark” mode (−0.9 V), the film was dark blue, blocking 92.6% of the total solar energy. The m‐WO_3−*x*_ NWs film can realize large Δ*T* of 93.2% (@633 nm), 91.7% (@800 nm), 88.5% (@1200 nm), and 86.8% (@1600 nm) in the voltage window of −0.9–0.7 V. In voltage range from 0.7 to −0.3 V, the NIR transmittance modulation was caused by the localized surface plasmon resonance (LSPR) effect due to Al^3+^ adsorbing onto m‐WO_3−*x*_ NWs. In voltage range from −0.3 to −0.5 V, NIR modulation is realized by LSPR and phase transition (from dielectric to metallic) caused by Al^3+^ insertion into m‐WO_3−*x*_ lattice, while VLT modulation is achieved by bandgap transitions (intra‐band and inter‐band transitions) under lower potential (from −0.5 to −0.9 V). The high charge density of Al^3+^ has allowed a smaller degree of intercalation/deintercalation, rendering fast switching kinetics (*τ*_c_/*τ*_b_ = 16 s/13 s), high coloration efficiency (254 cm^2^ C^−1^), and excellent cycle stability (5.5% capacity decay after 2000 cycles) of m‐WO_3−*x*_ NWs films. Guo et al. discovered that amorphous WO_3_ (a‐WO_3_) films realized larger Δ*T* (≈63.0%) and higher coloration efficiency (72.0 cm^2^ A^−1^) in Al^3+^‐based electrolyte than in Li^+^/Na^+^‐based electrolytes.^[^ ^63^ ^]^ Upon repeated ion intercalation/deintercalation, Li^+^ will be accumulated and difficult to be extracted, large Na^+^ will cause the destruction of ion‐diffusion channels due to excessive lattice expansion, while Al^3+^ intercalation/deintercalation is highly reversible. The superior Al^3+^‐induced electrochromic performance of a‐WO_3_ is ascribed to less amount of ion trapping, lower degree of lattice expansion, crystal structure stabilization due to the Coulombic interaction between Al^3+^ and the host, and the shallow diffusion depth of Al^3+^.

An inorganic heterojunction–based electrochromic nanostructured TiO_2_/MoO_3_ was also prepared for Al^3+^‐based electrochromism.^[^ ^64^ ^]^ The wide lattice spacing of MoO_3_ and built‐in electric field within the heterostructure have enabled fast and reversible pseudocapacitive Al^3+^ intercalation, rendering large Δ*T* (54%), fast switching time of ≈1 s, high coloration efficiency of 128 cm^2^ C^−1^ and excellent cyclic stability with 91.4% optical modulation retention, superior than those in Li^+^/Na^+^‐based electrolytes. Tang et al. also evaluated the effect of anions on Al^3+^ intercalation in sputtered WO_3_ film and found out that a proper ratio of Cl^−^/NO_3_^−^ (9:1) leads to larger Δ*T* (73.4%@650 nm) and higher coloration efficiency of 120.8 cm^2^ C^−^ in WO_3_‐based AECD, which was ascribed to the NO_3_^−^‐assisted de‐solvation of Al^3+^ for the stable embedding and shedding of Al^3+^ in WO_3_ film.^[^ ^65^ ^]^

#### 2.1.3 Other Multivalent‐Ions (Ca^2+^, Mg^2+^)‐Based Multivalent Electrochromism

**Figure 3:**
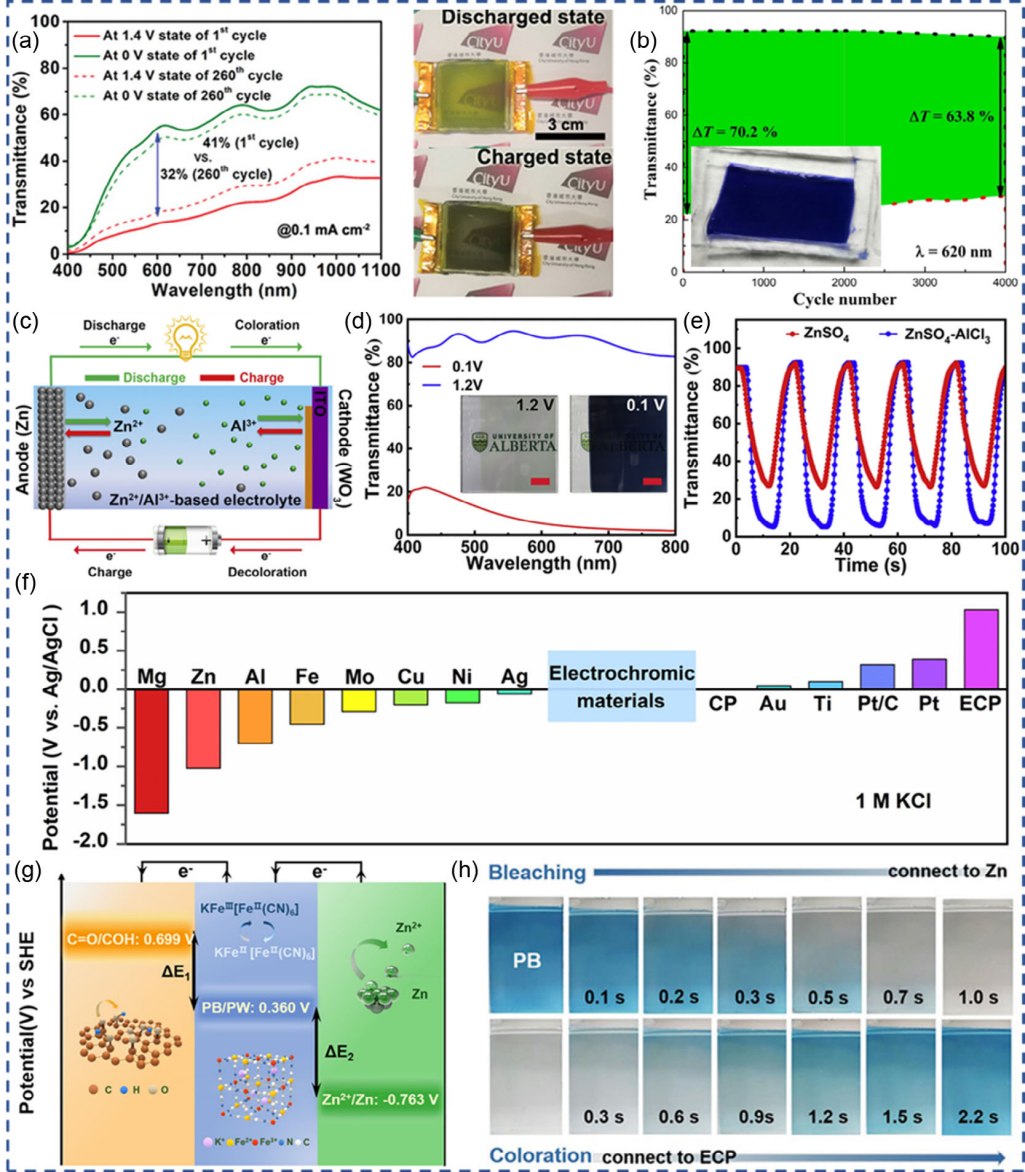
a) Transmittance modulation of the calcium‐ion‐based electrochromic battery (CIEB) at different cycles, and photos of the CIEB at discharged and charged states. Reproduced with permission.^[^ ^21^ ^]^ Copyright 2021, Wiley‐VCH. b) Cycling stability of a Mg‐anode‐based ECD with two WTO‐8 electrodes. Inset is the device at colored state. Reproduced with permission.^[^ ^46^ ^]^ Copyright 2023, Elsevier. c) Schematic of the rechargeable aqueous Zn^2+^/Al^3+^ electrochromic battery, d) transmittance spectra and photographs (scale bar 1 cm) of WO_3_ in ZnSO_4_–AlCl_3_, e) dynamic transmittance (@632.8 nm) of WO_3_ in ZnSO_4_–AlCl_3_ and 1 m ZnSO_4_. c–e) Reproduced with permission.^[^ ^73^ ^]^ Copyright 2019, Elsevier. f) Electrode potential of various materials in 1 m KCl electrolyte, g) energy‐level‐transition diagram of ECP, PB, and Zn, h) the complete bleaching/coloration process of WO_3_ connected with Zn and ECP. f–h) Reproduced with permission.^[^ ^77^ ^]^ Copyright 2022, American Chemical Society.

Aside from Zn^2+^ and Al^3+^, ion‐intercalation‐based ECDs employing other multivalent ions (e.g., Ca^2+^ and Mg^2+^) are also being explored. The significant advantage of Ca and Mg is that they can be uniformly deposited from electrolyte solution, and there is almost no dendrite formation. Ca^2+^ has the advantages of natural abundance, low cost, and stable valence state.^[^ ^66^ ^]^ The Ca^2+^ ion radius (0.99 Å) is similar to Na^+^ (1.02 Å), and slightly larger than Zn^2+^ (0.74 Å), Li ^+^ (0.76 Å), and Mg^2+^ (0.72 Å).^[^ ^30^, ^66^ ^]^ In addition, the charge density and polarization strength of Ca^2+^ are smaller than those of Al^3+^ and Zn^2+^, allowing improved diffusion kinetics and higher power density in Ca^2+^‐based electrochemical devices.^[^ ^30^, ^67^ ^]^ Wang et al. verified the conversion of WO_3_ into W and CaO caused by Ca^2+^ insertion. Ca^2+^ intercalation into the center site of the WO_3_ lattice were also imaged, yet only for ≈2 nm into the film, which may be attributed to the limited diffusivity of large Ca^2+^ ions in the dense W metal layers. This study demonstrates the atomic reaction process of Ca^2+^ intercalation, which is essential for ECDs.^[^ ^68^ ^]^ Doping was adopted to facilitate Ca^2+^ intercalation in WO_3_. Proper amount of Hf doping in WO_3_ (7% Hf‐WO_3_) was found out to render larger Δ*T* (75% @714 nm), higher coloration efficiency (161.87 cm^2^ C^−1^) and higher diffusion coefficient (2.33 × 10^−8^ cm^2^ s^−1^) in Ca^2+^ aqueous electrolyte, surpassing those in Li^+^ electrolyte.^[^ ^47^ ^]^ Ca–ECD was also assembled with water‐in‐salt Ca(OTF)_2_ electrolyte, vanadium oxide (VO_*x*_) and indium hexacyanoferrate (InHCF) films as anode and cathode, assembling a calcium‐ion‐based electrochromic battery (CIEB). As shown in **Figure** **3**a, the CIEB demonstrates large Δ*T* of 41.1%, good cycling stability (Δ*T* = 32% after 260 cycles), and a greenish yellow to black electrochromism.^[^ ^21^ ^]^

Mg^2+^ has higher resource abundance and lower reactivity than alkaline earth metal, endowing improved safety, and cost effectiveness in Mg^2+^‐based electrochemical devices. Mg^2+^ manifests similar ionic radius with Li^+^ (0.72 vs 0.76 Å) and a higher charge density than Li^+^.^[^ ^30^ ^]^ However, Mg^2+^‐based ECDs are currently hampered by limited charge transfer and solid‐state diffusion issues.^[^ ^29^ ^]^ Novák et al. demonstrated that bound lattice water can reduce the strong polarization effect of Mg^2+^, facilitating Mg^2+^ insertion into electrochromic metal oxide.^[^ ^69^ ^]^ Wang et al. reported the electrochemical intercalation of Mg^2+^ in WO_3_ and WO_3_·nH_2_O in a Mg^2+^ nonaqueous electrolyte. Due to the presence of structural water, WO_3_·nH_2_O has improved kinetics yet decreased capacity for Mg^2+^ storage. These results indicated that the electrochemical behavior of WO_3_ and WO_3_·nH_2_O in nonaqueous Mg^2+^ electrolyte can be regulated by structural water.^[^ ^70^ ^]^ Alternatively, doping is also effective in enabling Mg^2+^ electrochromism. W‐doping into TiO_2_ (WTO) can reduce the Mg^2+^ intercalation energy and enhance Mg^2+^ diffusion coefficient. With proper amount of W doping (W precursor 8%), WTO‐8 manifests larger Δ*T* and higher coloration efficiency in MgSO_4_ than in LiSO_4_. As a result, Mg–ECD fabricated by two WTO‐8 electrodes sandwiching a Mg electrode manifest excellent electrochromic reversibility and durability, exhibiting Δ*T* of 70.2% and 63.8% at the 1st and 4000th cycle (Figure 3b), respectively.^[^ ^46^ ^]^ Zhao et al. reported a nonstoichiometric tungsten oxide (W_18_O_49_) NWs‐based Mg^2+^ ECD with fast switching, high Δ*T*, and excellent stability. The electrochromic W_18_O_49_ NWs have a short coloration/bleaching time (2.7 s/3.6 s), high coloration efficiency of 116 cm^2^ C^−1^ and excellent cyclic stability in MgCl_2_ solution. The diffusion coefficient of Mg^2+^ is 5.29 × 10^−10^ cm^2^ s^−1^, which is higher than that of H^+^ and Al^3+^ in W_18_O_49_ NWs, explaining the favorable electrochromic performance enabled by Mg^2+^ insertion.^[^ ^71^ ^]^

#### 2.1.4 Hybrid Electrolyte for Multivalent Electrochromism

Although multivalent‐ion‐intercalation/deintercalation‐based ECDs have shown great potential and achievements have been made, there are still challenges to be overcome. Unlike monovalent ions, the intercalation/de‐intercalation of multivalent metal ions will cause serious cyclic stability degradation of ECMs, thus greatly shortening the service life of ECDs.^[^ ^72^ ^]^ Hybrid electrolytes containing dual‐metal ions have been adopted to improve the Δ*T*, switching kinetics, coloration efficiency, and cycling stability of ECMs/ECDs.

To improve the capacity and switching time of ECDs, and to solve the dynamic matching problem between different guest ions and host ECMs, a feasible solution is to use dual‐metal ions to provide multiple charges to increase the reaction rate.^[^ ^63^, ^73^ ^]^ As schemed in Figure 3c, Li et al. proposed an electrochromic battery using Zn^2+^/Al^3+^ hybrid electrolyte, electrodeposited WO_3_ cathode, and Zn anode.^[^ ^73^ ^]^ With lower intercalation barrier, Al^3+^ intercalation is responsible for the coloration of WO_3_ in hybrid electrolyte, while Zn^2+^ participates in the redox reaction on the Zn anode. Compared to Zn^2+^ electrolyte, such a hybrid electrolyte allows improved electrochemical and electrochromic performance in WO_3_ (Figure 3d,e), including larger Δ*T* (88%), fast switching kinetics (*τ*_c_/*τ*_b_ = 3.9 s/5.1 s), higher capacity (185.6 mAh m^−2^), and improved cycling stability (92% Δ*T* retention after 2500 cycles). Following this hybrid electrolyte concept, a Zn–PB ECD was also constructed using Zn^2+^/K^+^ hybrid electrolyte, rendering high Δ*T* (≈83% @632.8 nm), fast self‐bleaching (2.8 s), and fast switching speed (*τ*_c_/*τ*_b_ = 3 s/8.4 s).^[^ ^70^ ^]^ To overcome the issues of Li^+^ aggregation in amorphous tungsten oxide (a‐WO_*x*_) films and the slow transport kinetics of multivalent ions, Yu et al. also attempted an ECD based on nonaqueous Al^3+^/Li^+^ hybrid electrolyte.^[^ ^74^ ^]^ Al^3+^ is designated to provide high Δ*T* and improved stability, while Li^+^ allows faster switching (especially faster bleaching) due to its higher diffusion coefficient. With proper amount of Al^3+^ (10%), the Al^3+^/Li^+^ hybrid electrolyte rendered Δ*T* of 89.7%, fast switching kinetics (*τ*_c_/*τ*_b_ = 11.9 s/14.5 s), and much improved cycling stability in a‐WO_*x*_ films. As discussed in Section 2.1.1, the strategy of superimposing allows fabrication of multicolor ECDs. Two working electrodes (WO_3_ and the Ti–V_2_O_5_) were superimposed with Al frame in between, constructing a multicolor ECD with hybrid Li^+^/Al^3+^ electrolyte. Hybrid Li^+^/Al^3+^ electrolyte guarantees fast switching kinetics, higher capacity, and improved cycling stability, rendering multicolor (yellow, transparent, red, green, blue, and black) and improved capacity of 933 mAh m^−2^ in the as‐assembled ECD.^[^ ^72^ ^]^ Very recently, Liu et al. evaluated the electrochemical and electrochromic performance of Nb_18_W_16_O_93_ in various zinc‐based dual‐ion electrolytes (Zn^2+^–Al^3+^, Zn^2+^–Mg^2+^, and Zn^2+^–K^+^).^[^ ^75^ ^]^ With small ionic radii and high charge density of Al^3+^, the Zn^2+^–Al^3+^‐mixed electrolyte enabled an obviously superior specific capacity (160 mAh m^−2^), larger Δ*T* of 90.5%, and excellent cyclic stability (5000 cycles with 93.13% retention) in the Nb_18_W_16_O_93_ electrode.

#### 2.1.5 Potential Gradient‐Induced Multivalent Electrochromism

One of the advances of the aforementioned multivalent metal ions is their low reactivity in comparison to alkaline metals, allowing the construction of ECDs using metal electrodes.^[^ ^24^ ^]^ Owing to the relatively low‐standard redox potential of these metals (e.g., $E_{{\text{Zn}/\text{Zn}}^{2+}}$ = −0.76 V vs normal hydrogen electrode (NHE)),^[^ ^24^ ^]^ the potential gradient between the metal anode and electrochromic cathode can drive the thermodynamically favored spontaneous charge transfer, rendering “self‐coloration”^[^ ^24^ ^]^ or “self‐bleaching”^[^ ^76^ ^]^ in different ECMs.^[^ ^77^ ^]^ The spontaneous “self‐coloration” is realized in Zn–ECD with MTWO cathode in ZnSO_4_ aqueous electrolyte.^[^ ^24^ ^]^ Specifically, the redox potential difference (Δ*E*) between MTWO (>0.24 V vs standard hydrogen electrode (SHE)) and Zn (≈−0.76 V vs SHE) serves as the driving force to cause Zn oxidation (with Zn^2+^ ions formation) and MTWO reduction (with Zn^2+^ intercalation), when MTWO and Zn are connected. Accordingly, the reduced valance state of W^6+^, Mo^6+^, and Ti^4+^ would lead to the fast “self‐coloration” (coloration time 14 s) of MTWO with large Δ*T* of 76% (@632.8 nm).^[^ ^24^ ^]^ Wang et al. constructed an ECD with Al foil anode and PB cathode in KCl electrolyte.^[^ ^76^ ^]^ Short‐circuit of the cathode and anode allows fast “self‐bleaching“ of the PB cathode (Δ*T* of 52.2% @670 nm) due to Fe^3+^/Fe^2+^ reduction from PB to PW (Prussian white). Dissolved oxygen in aqueous electrolyte can slowly restore the blue color in the PB film or the PW film can be colored with electric bias at 2 V.^[^ ^76^ ^]^ Other metals are also adopted to assemble self‐bleaching ECDs with PB films.^[^ ^78^, ^79^ ^]^ For example, Zhai et al. reported an ECD using PB cathode and Mg metal anode, enabling the self‐bleaching of PB film.^[^ ^78^ ^]^ Tian et al. constructed a self‐bleachable ECD based on PB cathode and Ni anode in a mixed electrolyte of K^+^/Ni^2+^ (1:1), realizing Δ*T* of 39.45%.^[^ ^79^ ^]^

Huang et al. measured the redox potential (vs Ag/AgCl) of a series of materials in aqueous KCl electrolyte to screen suitable electrodes to pair with ECMs for self‐coloration and self‐bleaching.^[^ ^77^ ^]^ As shown in Figure 3f, metals (e.g., Al, Zn, Mg) normally have negative redox potential values, while Pt and etched carbon papers (ECPs) have positive redox potentials. Inspired by the large potential gradient (r*E*_1_ and r*E*_2_ in Figure 3g), Zn and ECPs are employed as the counter electrodes to drive the “self‐bleaching” and “self‐coloration” of PB, respectively. Larger potential difference endows faster switching kinetics. As shown in Figure 3h, the as‐constructed Zn–PB–ECP ECD allows fast “self‐bleaching” and “self‐coloration” within a few seconds when connecting Zn–PB and PB–ECP, respectively, demonstrating a sustainable ECDs needless of external power supply. Similar device structure applies for Zn–WO_3_–ECP and Zn–PEDOT–ECP.^[^ ^77^ ^]^ Following the same concept, Mg–PB–MnO_2_, Mg–WO_3_–MnO_2_, and Mg–PANI–MnO_2_ ECDs in organic electrolytes (Li^+^ in propylene carbonate) are also constructed with self‐coloration and self‐bleaching capability, demonstrating the universality of this “potential gradient” strategy in assembling energy‐efficient ECDs.^[^ ^80^ ^]^

It should be noted that the electrolytes used for these ECDs are not necessarily based on multivalent metal ions; however, there are accompanied redox reactions on the metal anode (e.g., Zn/Zn^2+^, Al/Al^3+^),^[^ ^76^ ^]^ thus these ECDs are also included for multivalent electrochromism.

Instead of using metal foil anodes, electrochromic anodes based on ion insertion/extraction can also be applied to construct “rocking chair” multivalent ECDs through the potential gradient between anode and cathode. For example, Zhang et al. constructed a dual‐mode electrochromic platform with “self‐coloring” and self‐bleaching” properties by sandwiching Zn between PB–WO_3_.^[^ ^81^ ^]^ Apart from using the redox potential difference between Zn and WO_3_/PB to give the device the ability to switch colors, the redox potential difference between WO_3_ and PB was further altered by using a KCl–ZnSO_4_ electrolyte system. Driven by the potential difference, K^+^ was extracted from the reduced WO_3_ and inserted into the PB electrode to realize the spontaneous color‐switching process.

### 2.2 Reversible Metal‐Electrodeposition‐Based Multivalent Electrochromism

**Figure 4:**
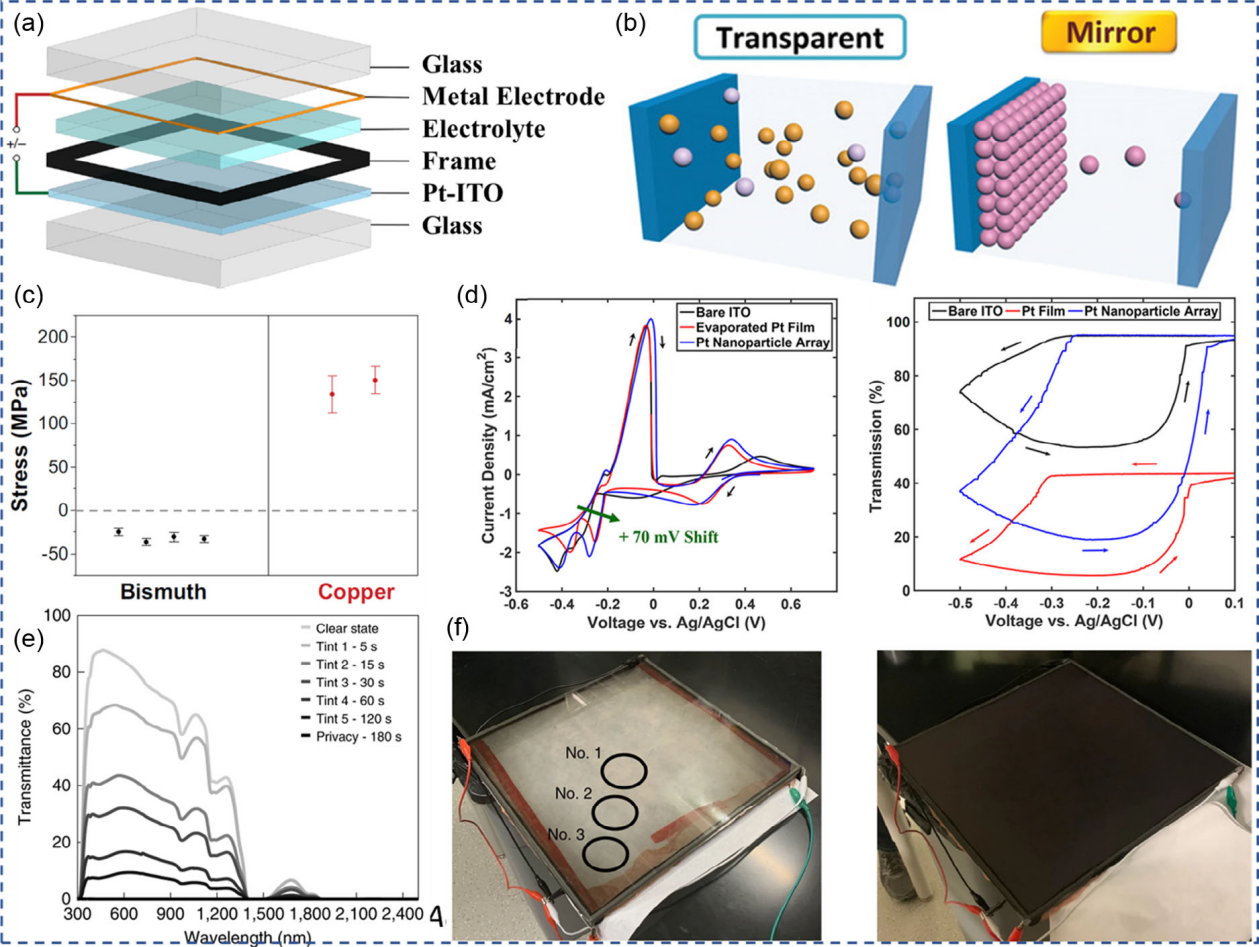
Electrochromism based on RME. a) Representative RME device configuration. Reproduced with permission.^[^ ^12^ ^]^ Copyright 2021, Springer Nature. b) Schematics of the working mechanism of RME device. Reproduced with permission.^[^ ^13^ ^]^ Copyright 2020, Wiley VCH. c) Stress in Bi‐ and Cu‐based RME films. Reproduced with permission.^[^ ^83^ ^]^ Copyright 2023, Wiley VCH. d) Cyclic voltammetry and corresponding transmission vs voltage curves of Bi–Cu electrolyte with bare indium tin oxide (ITO), Pt film ITO, or Pt–nanoparticle (NP)‐decorated ITO working electrodes, and Ag/AgCl reference electrode at a scan rate of 20 mV s^−1^. Reproduced with permission.^[^ ^90^ ^]^ Copyright 2018, American Chemical Society. e) Transmittance spectra of poly(vinyl alcohol) (PVA)‐added CuBi‐based RME device, and f) photograph of 927 cm^2^ device at transparent (left) and dark state (right). e–f) Reproduced with permission.^[^ ^12^ ^]^ Copyright 2021, Springer Nature.

Metals with high‐extinction coefficients are highly opaque at thickness of tens of nanometers, which are intriguing for dynamic window applications.^[^ ^15^, ^82^ ^]^ RME is an fascinating technology that are drawing attention in metal batteries (e.g., Li, Na, and Zn batteries) and dynamic glazings.^[^ ^83^ ^]^ Aside from the multivalent‐ion‐intercalation/deintercalation‐induced electrochromism, multivalent ECDs based on RME are also promising for thermal management and dynamic windows with color neutrality, fast switching, and durability.^[^ ^84^ ^]^ As schemed in **Figure** **4**a, generally this type of ECD is also assembled with working electrode and counter electrode (metal frame/metal mesh) sandwiching electrolyte in between.^[^ ^12^ ^]^ Under open circuit potential, multivalent metal ions (e.g., Cu^2+^, Bi^3+^, Zn^2+^, Ni^2+^) are dissolved in the electrolyte and the ECDs remain transparent as shown in Figure 4b.^[^ ^13^ ^]^ Upon discharging, the metal ions will be reduced and electrodeposited onto the working electrode, forming a dense and thin metallic film that reduces the transmittance (sometimes with notably enhanced reflectance) of the ECDs. Reversely, during charging, the electrodeposited metallic film will be oxidized and stripped from the working electrode, restoring the initial transparent state of ECDs.^[^ ^84^ ^]^ The overall electrochemical performance (e.g., large Δ*T*, fast response, coloration efficiency, uniformity, scalability, and durability) of RME‐based ECDs depend on many factors, including electrolyte composition, electrode modification, device configuration, and the mechanical stability of electrodeposited metal films. Attempts have been made adopting different electrolyte compositions with single‐ and dual‐metal ions to enhance the electrochromic performance of RME–ECDs, and the progress will be discussed in this section.

#### 2.2.1 Single‐Metal‐Ion‐Based RME ECDs

RME‐based ECDs have been demonstrated using Ag electrolytes decades ago, yet only with a small size of 1–4 cm^2^ and poor Coulombic efficiency and durability, not to mention the high cost of Ag.^[^ ^15^, ^85^ ^]^ Alternative metals, for example, Cu, Bi, Zn, Ni, etc., with cost effectiveness and high theoretical coloration efficiency are more viable for practical RME dynamic windows.^[^ ^15^ ^]^ Electrolyte composition is of great significance in determining the electrochromic performance of RME‐based ECDs. The effect of anions (NO_3_^−^, Br^−^, SO_4_^2−^, ClO_4_^−^, and Cl^−^) and pH values have been evaluated for Cu^2+^‐based aqueous electrolyte for RME.^[^ ^84^ ^]^ In acid‐free electrolytes, these anions are unfavorable for RME due to unwanted side reactions including irreversible cathodic reduction of NO_3_^−^, anodically formed yellow Br^3−^ complexes, formation of insoluble Cu_2_O species (in SO_4_^2−^, ClO_4_^−^ electrolytes) that reduce light transmission, and Cl^−^‐blocking Pt‐active sites. Pt NPs can improve the electronic conductivity and accelerate the transfer of electrons and ions by building Schottky barrier and strong internal electronic field,^[^ ^86^ ^]^ and are thus incorporated onto fluorine‐doped tin oxide (FTO) electrodes as will be discussed later. In acid electrolytes, SO_4_^2−^ will render fast etching of the ITO substrate, and halide anions will also etch the ITO substrate due to formation of In–X bonds, thus acidic ClO_4_^−^‐based electrolytes are screened as favorable aqueous electrolyte for Cu–RME ECDs. RME of Cu^2+^ and Bi^3+^ have been attempted in aqueous acidic ClO_4_^−^ electrolytes, the residual stress in electrodeposited Cu and Bi films are quantified as presented in Figure 4c.^[^ ^83^ ^]^ Despite the excellent electrochromic performance of Cu–RME dynamic windows in acidic ClO_4_^−^ electrolytes, electrodeposited Cu films suffer from limited rest stability due to the tensile stress and stress‐corrosive cracking, leading to fracture and delamination of the electrodeposited Cu film within 24 h soaking in electrolyte. On the contrary, electrodeposited Bi with compressive stress can sustain a longer rest stability for more than 9 weeks. Other metal films with compressive stress are also predicted with favorable rest stability. To harvest the high ionic conductivity of aqueous electrolytes and large voltage window of deep eutectic solvents (DESs), a hybrid electrolyte consisting of water and DES was also formulated for Cu–RME, enabling a durable RME electrochromic electrode with Δ*T* of ≈50% and cycling stability of 5000 cycles (Δ*T* degradation of 4.71%).^[^ ^23^ ^]^ Aside from electrolyte composition, device configuration also affects the performance of RME‐based ECDs. A PB‐based counter electrode was introduced to pair with Bi–RME working electrode and facilitate transverse ion migration, enabling the construction of a 25 cm^2^ ECD with intermediate blue color, large Δ*T* of 67.5% (@700 nm), and fast coloration within 3 s.^[^ ^87^ ^]^

Acidity is needed for Bi‐based RME ECDs to avoid formation of insoluble Bi(OH)_3_ precipitates, yet low pH will affect the shelf life of ECDs due to etching of ITO.^[^ ^82^ ^]^ As a result, pH neutral electrolytes (zinc acetate) are also considered for RME ECDs, allowing construction of 100 cm^2^ dynamic windows (with zinc mesh counter) with large ΔT of 80% (@600 nm) within 30 s.^[^ ^82^ ^]^ However, different ratios of ZnO and Zn(OH)_2_ are formed on the working electrode at different stages, affecting the reversibility of the ECDs. Other electrolyte compositions are also attempted to evaluate the effect of carboxylates chain length, the halogen in haloacetate, supporting halides, and non‐coordinating anions on the reversibility of Zn‐RME.^[^ ^88^ ^]^ It was screened that electrolyte composed of ZnCl_2_–ZnBr_2_–Na formate and ZnCl_2_–ZnSO_4_–HCOONa manifests high Coulombic efficiency and fast switching kinetics; however, ECDs based on these electrolyte formulations still suffer from limited cycling stability due to Zn(OH)_2_ accumulation.^[^ ^88^ ^]^

#### 2.2.2 Dual‐Metal‐Ions‐Based RME ECDs

Problems preserve for single‐metal‐based RME devices, including production of dendrite‐like particles with numerous protrusions, high transmission at colored state, and difficulty for complete metal stripping during oxidation. Nonetheless, dual‐metal (or alloy)‐based RME can achieve facile deposition, rapid response, improved durability, high Δ*T*, large area device, and other excellent performance, by electrode modification and electrolyte optimization. Previous single‐metal‐based RME have been studied and reported, with Bi being more explored for electrochromic applications. It was found out that a Bi–Cu electrolyte is favorable for RME. Cu alone is not suitable for RME applications because of its red color, but Bi makes for an excellent complementary metal for its strong absorbing black color, and similar electrochemical potential to that of Cu.^[^ ^89^ ^]^ Therefore, RME–ECDs based on Bi–Cu electrochemical co‐deposition have been assembled, adopting various optimization methods as described later.

##### Modification of the Working Electrode

ITO and FTO are the most used working electrodes with high transparency and electronic conductivity, but the nucleation of metal electrodeposits is often difficult and nonuniform because of the heterogeneous surface chemistries of these substrates, restraining the cycle life of RME devices. Barile et al. introduced Pt‐NPs anchored by a self‐assembled monolayer of 3‐mercaptopropionic acid to achieve uniform metal electrodeposition by promoting nucleation across the ITO electrode.^[^ ^15^ ^]^ Moreover, Hernandez et al. introduced Pt‐NPs on the working electrode to facilitate metal nucleation, while maintaining a high transmittance and electronic conductivity.^[^ ^90^ ^]^ As shown in Figure 4d, it was demonstrated that Pt‐modified working electrode can reduce the nucleation overpotential by 70 mV compared with the unmodified working electrode, thus realizing larger optical contrast within the same voltage window. In addition, during electroplating/stripping, the nonuniform current density across the ITO/FTO will cause voltage drop from the edge to the center, especially for large‐area electrodes. Pt‐NPs‐decorated ITO/FTO electrodes can mitigate the voltage drop and achieve a larger area device assembly. Islam et al. have achieved selective electrodeposition using Pt‐NPs‐patterned ITO.^[^ ^91^ ^]^ It was found out that Pt‐NPs‐decorated area allows more uniform electrodeposition. Traditional RME dynamic windows use a Pt‐modified working electrode and a metal foil counter electrode, yet with unsatisfactory color switching speed and difficulty to reach colored state with low transmittance. The use of metal frame counter electrodes allows construction of dynamic windows with two working electrodes. RME–ECDs based on two pieces of parallel Pt‐modified working electrodes sandwiching a metal frame counter electrode were assembled. Compared to conventional device configuration (one working electrode, *T*% = 30% at colored state), this “two working electrodes” strategy allows RME on both working electrodes, rendering superior contrast ratios with lower transmittance of 15% at colored state. Unfortunately, this “two working electrodes” design will cause increased cost.

##### Modification of the Counter Electrode

**Figure 5:**
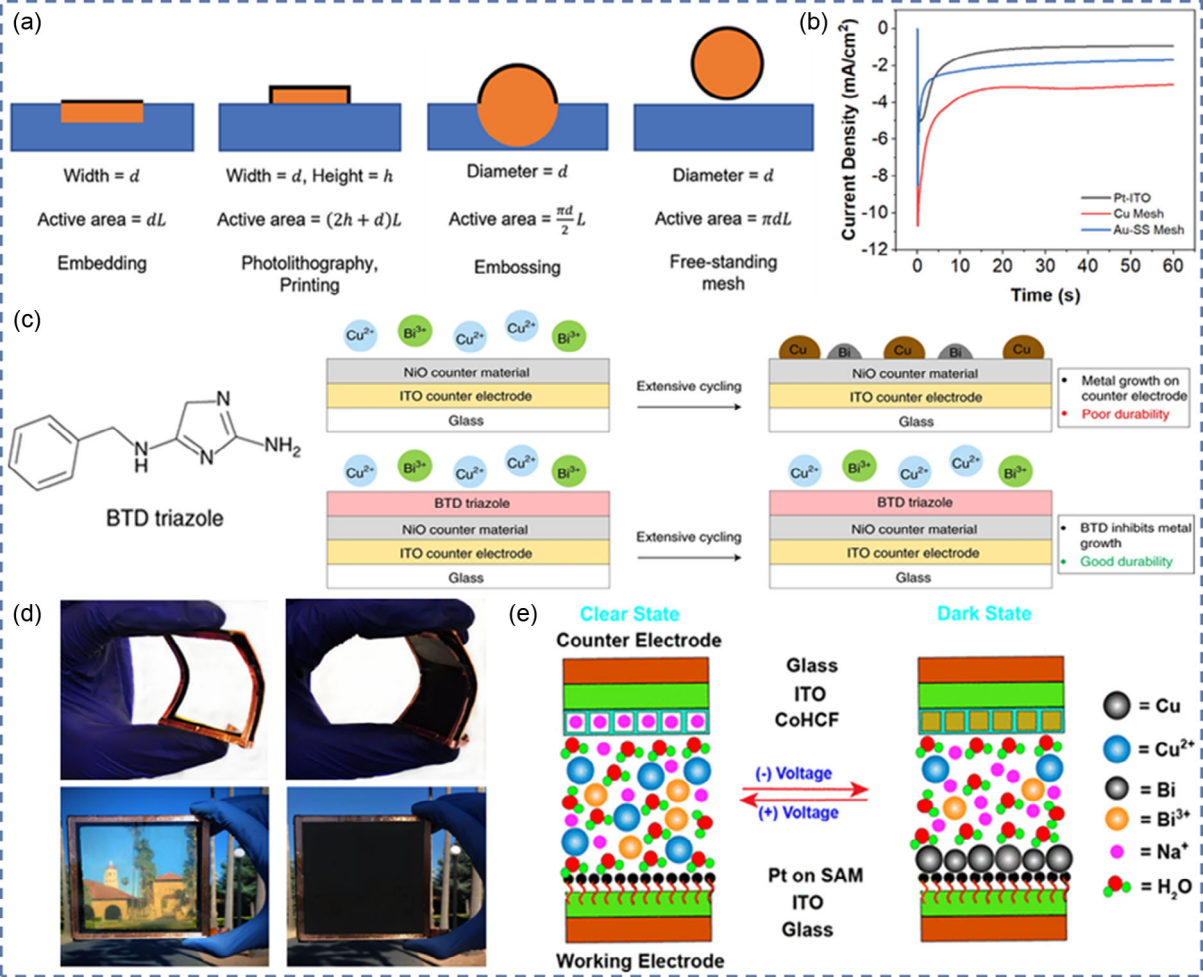
Modification of the counter electrode for ECDs based on RME. a) Metal mesh with various geometry, b) areal current density vs time of Pt‐ITO, Cu mesh, and Au‐coated stainless‐steel mesh for metal deposition induced at −0.7 V (vs Ag/AgCl) in Cu–Bi electrolyte. (a,b) Reproduced with permission.^[^ ^92^ ^]^ Copyright 2022, Wiley‐VCH. c) Schematic of N_5_‐benzyl‐1H‐1,2,4‐triazole‐3,5‐diamine‐ and LiNiO_*x*_‐modified counter electrode that ensures good durability in CuBi‐based dynamic window, and d) photographs of the assembled 25 cm^2^ flexible dynamic window and 100 cm^2^ dynamic window. c,d) Reproduced with permission.^[^ ^14^ ^]^ Copyright 2019, Springer Nature. e) Schematic of a hybrid dynamic window that incorporated CoHCF‐coated ITO glass counter electrode. Reproduced with permission.^[^ ^93^ ^]^ Copyright 2020, American Chemical Society.

Although Pt‐NPs‐decorated working electrode helps in mitigating surface inhomogeneity, the counter electrode limits the durability of the devices. Yeang et al. developed a woven mesh incorporating stainless‐steel (SS) core and Au‐capping layer to assist Cu–Bi plating.^[^ ^92^ ^]^ As shown in **Figure** **5**a, the transparency of the woven mesh can be adjusted by geometric design (line width and line spacing). For example, SS grid with a large line spacing has significantly reduced diffuse transmission and haze. This allows construction of large area device without significantly affecting the transparency. The areal current densities of Cu–Bi plating were compared for Pt–ITO (flat surface), Cu mesh (cylindrical line), and Au‐coated SS grids (cylindrical line)‐based counter electrodes. With a smaller fraction (75% for Pt–ITO and 37% for Cu mesh and Au–SS grids) of active area, Cu mesh and Au–SS grids enabled higher current response than the planar Pt–ITO due to the cylindrical geometry (Figure 5b). Islam et al. developed a dynamic window using insertion‐based planar LiNiO_*x*_ counter electrode, which promote transverse ion diffusion.^[^ ^14^ ^]^ Dynamic window with area of 25 cm^2^ were constructed using Pt‐NPs‐modified ITO working electrode and an electrodeposited porous LiNiO_*x*_ (≈200 nm thick)‐coated ITO counter electrode. As shown in Figure 5c, a 450 nm thick layer of N_5_‐benzyl‐1H‐1,2,4‐triazole‐3,5‐diamine was spin‐coated on NiO as the metal inhibitor to protect the NiO. Eventually, a 100 cm^2^ dynamic window was assembled, realizing fast response between clear and black states, realizing Δ*T* of 65% within a minute (Figure 5d). This suggests that proper design of counter electrode can assist CuBi RME in dynamic windows. Similarly, cobalt hexacyanoferrate (KCo[Fe(CN)_6_], CoHCF)‐coated ITO was also incorporated as the counter electrode to build a color‐neutral hybrid dynamic window, which manifest six times faster bleaching than the NiO‐based hybrid dynamic window, further emphasizing the effectiveness of counter electrode modification in facilitating Cu–Bi RME (Figure 5e).^[^ ^93^ ^]^

##### Electrolyte Engineering

Aside from electrode modification, electrolyte optimization is also of great importance in facilitating CuBi RME. Adding a small amount of polymer inhibitors can effectively prevent the dendrite formation. The presence of the polymer can disperse the spatial charges over the electrode surface, leading to uniform electrodeposition. Strand et al. added a small amount of poly(vinyl alcohol) (PVA) to the Bi–Cu electrolyte, obtaining a smooth and dense electrodeposition layer.^[^ ^12^ ^]^ As shown in Figure 4e,f, this PVA‐based polymer inhibitor allows VLT as low as less than 0.001%, and the assembly of a large area (>900 cm^2^) dynamic window with rapid, uniform coloration, and improved durability. In addition to electrochemical performance modulation, durability of dynamic windows under harsh environment can also be enhanced by electrolyte engineering. Antifreezing electrolyte is needed for low‐temperature operation due to the possible glass breakage caused by electrolyte expansion under freezing. Alcaraz et al. used hydroxyethyl cellulose as a coagulant and different alcohol‐based antifreeze agent (i.e., methanol, ethylene glycol, and glycerol) to increase the temperature durability of Cu–Bi RME‐based dynamic window.^[^ ^94^ ^]^ Without antifreezing agent, the dynamic window failed at temperatures <−2 °C due to electrolyte freezing. In comparison, dynamic window with 60% ethylene glycol and 40% water can be switched normally and reversibly at temperature as low as −40 °C, yet with hindered kinetics due to the bonding between alcohol groups and Cu^2+^. More favorable antifreeze agents are to be explored to optimize the electrolyte in future RME dynamic windows. The ratio of metal cations in the electrolyte will also affect the RME. Hernandez et al. found out an optimal electrolyte composition of Bi:Cu = 1:3.^[^ ^89^ ^]^ Higher Cu^2+^ concentration will lead to CuBr precipitation and thus affecting the metal stripping, while low‐Cu^2+^ concentration will hinder the galvanic displacement of Bi by Cu^+^. Cu–Bi electrolytes are often acidic to avoid the generation of insoluble Bi(OH)_3_, but the slow degradation of ITO in acidic solution increases the electronic resistance, resulting in slower switching speed and limited Δ*T*. In addition, hydrogen evolution often emerges in the acidic electrolyte. Therefore, Miller et al. explored alkaline electrolytes to improve the durability of dynamic windows.^[^ ^95^ ^]^ Hydrochloric acid and sodium hydroxide were used to formulate alkaline electrolyte with pH of 8.5, ethylene diamine tetraacetic acid was also added to dissolve Bi^3+^ by chelation under alkaline conditions. As a result, the ITO electrode maintained a stable sheet resistance over 10 days in alkaline electrolytes. Electrolytes based on different halides were also evaluated and it was revealed that iodine‐containing electrolytes triggered complete RME because I_2_ facilitated metal stripping. These optimizations of Bi–Cu electrolytes have led to the tremendous development of CuBi‐based RME dynamic windows.

A bistable Cu–Bi RME dynamic window normally presents three color states (transparent, grey, and black), RME devices owning multicolor states have significant advantages for radiant energy control. Zhao et al. reported an RME–ECD with five optical states by applying step potential and potentiostatic methods.^[^ ^96^ ^]^ By controlling the applied voltage and duration, bimetallic films with different morphology and metal ratio were obtained, rendering multicolor state associated with LSPR effect. The Bi and Cu ratios were quantified to be 64:36, 35:65, 74:26, and 39:61 in the purple transparent, purple mirror, yellow transparent, and yellow mirror states, respectively. The switching speed, cycling performance, and thermal resistance of this multicolor Bi–Cu RME–ECD are also superior, which further expands the application of CuBi‐based RME electronic devices.

Although Bi‐based black ECD has been extensively studied, it is still difficult to achieve a very low transmittance below 5% in the VIS light region even with the alloying strategy. Halides play a huge role for RME in both aqueous and nonaqueous electrolytes, due to the improved electrochemical kinetics brought by halide stabilization of the Cu (I) intermediate during deposition and dissolution.^[^ ^97^ ^]^ Thus, Cu halide electrolytes are also favorable for RME, but the red color of copper restrains its optical applications, and a single cation Cu halide electrolyte is not completely reversible. To address these issues, adding a second redox‐active metal ion aside from Bi has been attempted. Due to the high theoretical chromogenic efficiency and low oxophilicity of Pb, Barile et al. formulated electrolyte consisting of Pb(ClO_4_)_2_, CuCl_2_, Cu(ClO_4_)_2_, and LiClO_4_.^[^ ^15^ ^]^ It was demonstrated that the Cu–Pb electrolyte had excellent reversibility (RME over 4000 cycles with Δ*T* more than 60%). However, the toxic Pb should be carefully handled, thus nontoxic Ag and Au were also introduced as a second redox‐active metal ion. CuAg‐based RME shows reversible electrochemistry and comparable coloration efficiency to WO_3_. However, the solubility of Ag halide in aqueous electrolyte is often limited.^[^ ^15^ ^]^ Barile et al. demonstrated that the addition of gold ions to the aqueous electrolyte (containing CuCl_2_, NaAuCl_4_, NaCl, and NaBr) increased the optical reversibility of RME.^[^ ^98^ ^]^ The concentration of Br^−^ in the electrolyte is significantly lower than that of Cl^−^, avoiding the formation of dark red AuBr^4−^ to prevent the loss of electrolyte transparency. Formation of Cu–Au alloy was verified by comparing the cyclic voltammograms in electrolytes of Cu, Au, and Cu–Au. However, RME device with Cu–Au electrolyte suffers from inefficiency, unknown structure, and composition of the Cu‐Au mixture species, in addition to the high price of gold. It is realized that Ni is also an excellent material for RME because of its superior absorption capability in the solar spectral region, low cost, the same crystal structure (center cube), and highly similar lattice parameters with Cu (Ni: 0.353 nm and Cu: 0.361 nm). Barile et al. studied the electrochemical properties of Ni–Cu electrolyte in detail, and demonstrated that the amino sulfonate contributes to the EC behavior observed in Ni–Cu electrolytes.^[^ ^99^ ^]^ Guo et al. reported Ni–Cu‐based RME and reached optimum Coulombic efficiency and coloration efficiency at Ni:Cu ratio of 2:1 in dimethylsulfoxide solvent.^[^ ^100^ ^]^ Addition of PVA in Ni–Cu electrolyte system can also effectively inhibit dendrite growth, and the formation of NiCu alloy is verified by X‐ray photoelectron spectroscopy. A 2.5 × 5 cm device with rapid switching time (*τ*_c_/*τ*_b_ = 6.2 s/13.2 s) was thus assembled, blocking the NIR region at colored state. The alloy element Sn, as an electrochemical medium, also facilitates the electrodeposition and dissolution of Cu. A quasi‐solid‐state CuSn‐based RME device with enhanced cycle stability was implemented by Eh et al.^[^ ^13^ ^]^ The CuSn electrolyte manifest high ionic conductivity, thus essentially facilitating the RME of CuSn alloy. Furthermore, the as‐deposited CuSn film have higher resistance to surface oxidation, resulting in higher reflectivity of CuSn alloy films (compared to Cu) during prolonged air exposure. With a PVA‐based polymer electrolyte, the CuSn RME device also achieves an extended memory effect due to the impeded dissolution of CuSn alloy.

To achieve commercial meter‐scale RME dynamic windows, future studies are to be carried out on achieving low‐sheet resistance working electrodes to reduce the potential differences between the edge and center of the window, optically transparent metal–mesh‐based counter electrodes to reduce ion‐diffusion length, and optimized electrolyte composition with fast kinetics, high reversibility, and antifreezing capability.

### 2.3 DMLI‐Based Multivalent Electrochromism

Metallo–organic materials with multiple colors are also one of the typical ECMs. The different oxidation states of the metal centers can affect the metal‐to‐ligand‐charge transfer (MLCT), thus rendering reversible coloration/bleaching in metallo–organic materials, for example, polypyridyl complexes.^[^ ^16^, ^17^ ^]^ However, the electrochromism of these metallo‐organic materials are normally electrochemically activated in monovalent‐ion‐based electrolyte (normally Li^+^ or Na^+^).^[^ ^16^, ^101^ ^]^ Unlike MLCT‐associated electrochromism, metal–ligand coordination/dissociation can also be adopted for ECDs. The diverse option of metal ions has allowed dynamic metal–ligand coordination (DMLI) with various molecules. DMLI as one kind of supramolecular bond has intermediate strength and excellent reversibility.^[^ ^102^ ^]^ Chemical structure engineering of the ligand as well as alteration of metal ions have been found to create a spectrum of colors due to tunable absorption spectra.^[^ ^102^ ^]^ Based on the DMLI mechanism, rewritable papers employing molecules with tridentate groups have been developed, which can be written (colored) with Fe^2+^ and erased (bleached) by F^−^ because the metal‐F^−^ bond can dissociate the metal–ligand coordination.^[^ ^102^ ^]^

**Figure 6:**
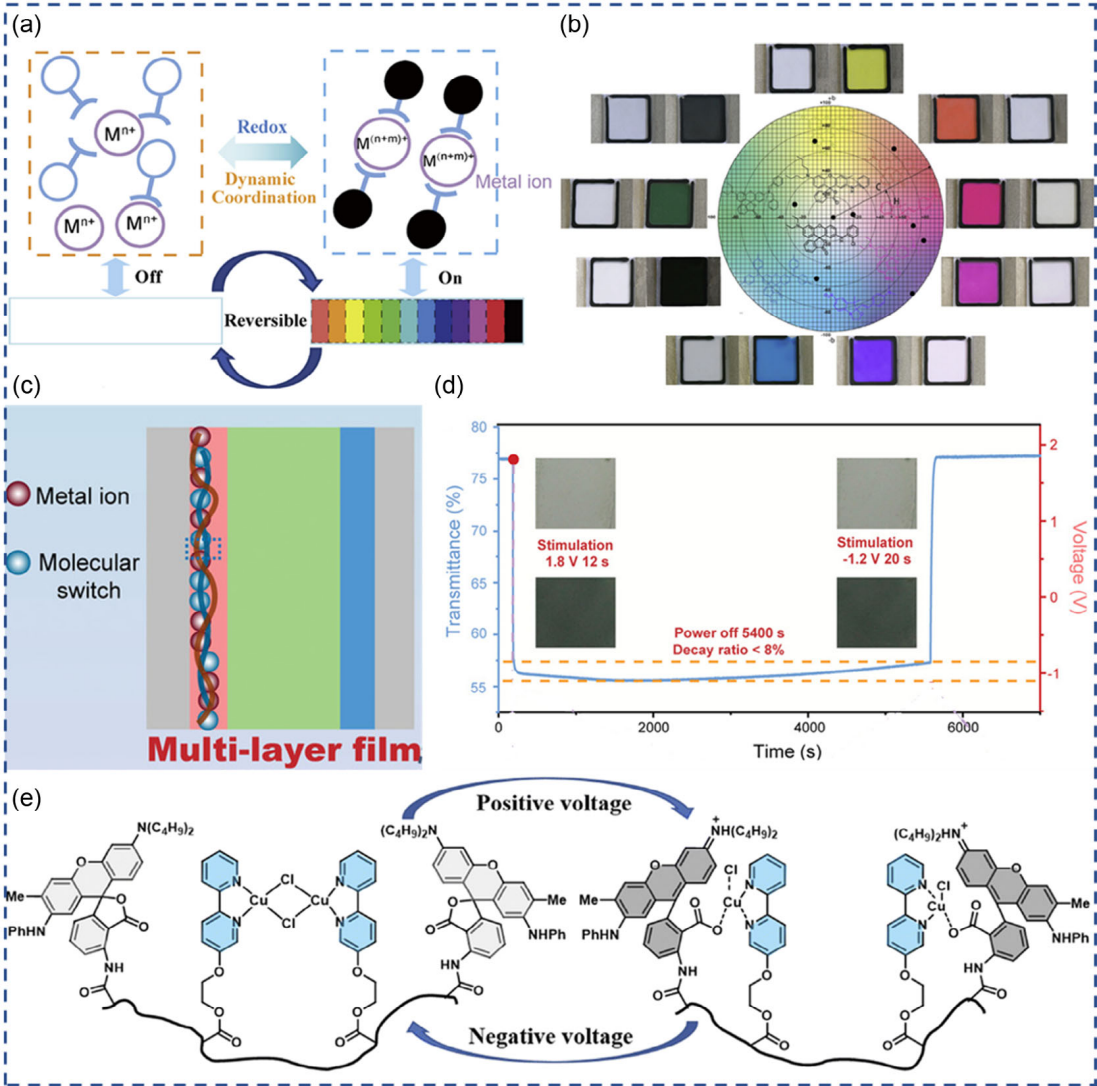
Electrochromism based on DMLIs. a) Working mechanism illustration of DMLI‐based electrochromism, and b) demonstration of DMLI EC device employing different molecular switches. a,b) Reproduced with permission.^[^ ^11^ ^]^ Copyright 2021, Elsevier. c) Schematics of bistable DMLI‐based EC devices, d) demonstration of bistability. c,d) Reproduced with permission.^[^ ^103^ ^]^ Copyright 2022, Wiley‐VCH. e) Bistable DMLI‐based EC device achieved by copolymer complex. Reproduced with permission.^[^ ^104^ ^]^ Copyright 2022, Royal Society of Chemistry.

Inspired by the multicolor enabled by DMLI, Zhang et al. designed unconventional DMLI‐based ECDs through dynamic coordination/dissociation between valence‐changing metal ions and switchable dyes.^[^ ^11^ ^]^ The configuration of DMLI‐based ECDs is quite similar to that of RME‐based ECDs, with two pieces of ITO electrodes sandwiching the electroactive electrolytes. As schemed in **Figure** **6**a, when the valence state of metal ions are changed (M^*n*+^ to M^(*n*+*m*)+^) by electric pulses, M^(*n*+*m*)+^ can coordinate with molecular switches, rendering colored states. Reversed bias can reduce the metal ions and dissociate the metal–ligand bonds, restoring transparency in the ECDs.^[^ ^11^ ^]^ Considering the selectivity, coordination ability, contrast ratio, and electrochemical stability of molecular switches and the safety, valence states, and redox potential of metal ions, DMLI based on black dye M1 (ODB‐2,2‐anilino‐6‐(dibutylamino)‐3‐methylfluoran) and Cu^+^/Cu^2+^ was screened out with feasibility for ECDs. Electrically oxidized Cu^2+^ can induce dynamic tautomerization of M1, with color changes from colorless to black. With optimized electrochromic solution formulation (concentration, anions, solvents, molar ratios between Cu^+^/M1, etc.), a multivalent ECD was assembled by two pieces of ITO sandwiching the CuI–M1 (1:4) electrolyte, realizing excellent electrochromic performance with high coloration efficiency (506.67 cm^2^ C^−1^) and good cycling stability (Δ*T* decay less than 10% after 2000 cycles). Color tunability was also realized as displayed in Figure 6b, multicolor ECDs were obtained by adopting different switchable dyes (M1–M9).^[^ ^11^ ^]^ However, these liquid ECDs suffer from limited bistability due to the undesirable thermal diffusion of ions and molecules.^[^ ^103^ ^]^ Bistability of ECDs refers to their capability of maintaining the transmittance at bleached and colored state without additional energy consumption (under open‐circuit potential).^[^ ^103^ ^]^ Aiming at improving the bistability of DMLI–ECDs, as schemed in Figure 6c, the switchable dyes (ODB‐2) are grafted on to polymer skeleton (ODBMA) and Cu(I) ions are also coordinated with polymers containing pyridine groups (PyMA–CuCl). After optimizing the content of Cu^+^ in PyMA–CuCl (14.3%), content of ODBMA (66.6%), thickness of the EC layer (0.63 μm), and composition of the ion‐storage layer, a solid ECD was assembled with excellent bistability and high coloration efficiency (112.2 cm^2^ C^−1^). As shown in Figure 6d, the solid ECD can maintain the colored state for 1.5 h with minimal transmittance variation without power supply.^[^ ^96^ ^]^ In addition, as shown in Figure 6e, copolymers containing both the cuprous complex and switchable dyes were designed and synthesized. The as‐assembled ECD manifests further improved bistability, after resting for ≈2.3 h (8300 s), there is only 10% transmittance variation at colored state.^[^ ^104^ ^]^

## 3 Application of Multivalent Electrochromism

**Figure 7:**
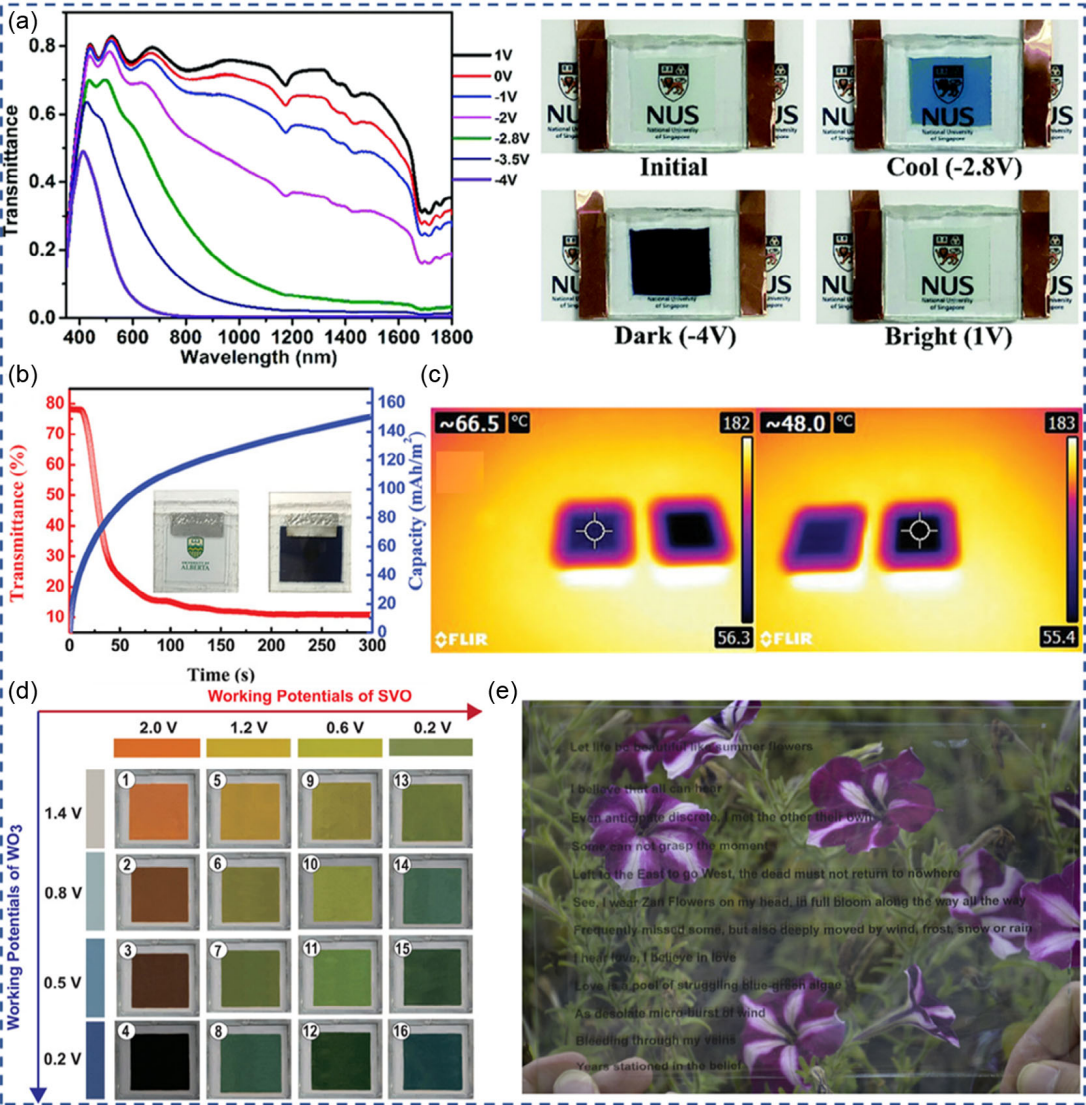
Demonstration of multivalent ECD applications. a) Dual‐band smart windows based on Al^3+^ intercalation in monoclinic WO_3−*x*_ NWs. Reproduced with permission.^[^ ^5^ ^]^ Copyright 2018, Elsevier. b) Energy‐storage ECDs with Zn–tungsten–molybdenum oxide (Zn–MTWO). Reproduced with permission.^[^ ^24^ ^]^ Copyright 2019, Wiley‐VCH. c) Thermal images of electrodeposited CuSn/fluorine‐doped tin oxide (FTO) and bare FTO on a hotplate. Reproduced under the terms of the CC‐BY Creative Commons Attribution 4.0 International license (https://creativecommons.org/licenses/by/4.0).^[^ ^13^ ^]^ Copyright 2020, The Authors, published by Wiley‐VCH. d) Multicolor transmissive electrochromic displays by superimposing SVO and WO_3_ layers. Reproduced with permission.^[^ ^36^ ^]^ Copyright 2022, Wiley‐VCH. e) See‐through electrochromic display. Reproduced with permission.^[^ ^11^ ^]^ Copyright 2021, Elsevier.

The rich electrochemistry of multivalent metal ions has brought new possibilities in ECDs. With different electrochemical mechanism, multivalent ECDs have found applications in smart window, energy storage, thermal management, multicolor display, and non‐emissive see‐through display as exemplified in **Figure** **7**.

### 3.1 Smart Windows

Smart windows capable of light and heat control are intriguing for green buildings. It is estimated that smart windows can increase worker productivity by 2% with optimal temperature and lighting control, and render 10–20% energy saving in buildings by reducing heat, ventilation, and air‐conditioning energy consumption.^[^ ^2^, ^83^, ^84^ ^]^ Commercial electrochromic smart windows have been developed by Sage,^[^ ^105^ ^]^ View,^[^ ^106^ ^]^ Halio,^[^ ^107^ ^]^ etc., mostly based on vacuum‐deposition technology, hindering the scalability and wide adoption of electrochromic smart windows. Solution‐processed multivalent smart windows have been achieved in both ion intercalation and RME‐based ECDs. As shown in Figure 7a, Al^3+^ intercalation enabled construction of dual‐band smart windows with monoclinic oxygen deficient WO_3−*x*_ NWs working electrode and ITO counter electrode. This smart window can independently adjust the VIS and NIR transmittance by altering the voltage, thus offering modes of transparent/bright, cool, and dark.^[^ ^5^ ^]^ Similarly, dual‐band smart windows are also assembled with two PANI electrodes sandwiching Zn frame in between, offering bright, cool, and dark states for smart window applications.^[^ ^42^ ^]^ RME‐based smart windows have the advantages of color neutrality, large optical modulation, and fast switching, although problems including durability, uniformity, and rest stability persist.^[^ ^15^ ^]^ With Pt‐modified ITO working electrode and optimal electrolyte formulation (Cu–Bi ions with PVA added), large‐area 927 cm^2^ RME‐based ECDs were assembled, reaching extremely low transmittance (*T* = 0.001%) across a wide wavelength range (300–2400 nm), color neutrality (chroma <5) and large‐solar‐heat‐gain coefficient (SHGC) tunability. The ECD realizes rSHGC of 0.56, surpassing those existing smart windows by Sage and View.^[^ ^12^ ^]^

### 3.2 Energy Storage

Multivalent‐ion‐intercalation‐based ECDs are endowed with energy‐storage capability, as the coloration/bleaching processes are realized through charge storage/release. However, it should be noted that there is always a trade‐off between energy storage and electrochromic performance, as higher charge‐storage capability is pursued for energy storage while higher coloration efficiency (less charge consumption) is required for electrochromics. Energy‐storage capability has been reported for multivalent‐ion‐intercalation‐based ECDs. For example, as shown in Figure 7b, when the MTWO–Zn ECD is self‐colored, a capacity of 150 mAh m^−2^ can be harvested with simultaneous Δ*T* of ≈70%, demonstrating electrochromic‐energy‐storage dual functionality.^[^ ^24^ ^]^ Other examples of ion‐intercalation‐based electrochromic‐energy‐storage multivalent ECDs include but not limited to Zn–NiO (17.1 mAh m^−2^),^[^ ^108^ ^]^ CIEB based on VO_*x*_ and InHCF (energy density of 51.4 mWh m^−2^ at power density of 1737.3 mW m^−2^),^[^ ^21^ ^]^ WO_3_–Zn in Zn^2+^–Al^3+^ electrolyte (185.6 mAh m^−2^),^[^ ^73^ ^]^ etc. Similarly, multivalent ECDs based on RME and DMLI should be able to store energy due to the accompanied charge‐transfer processes. However, energy‐storage function is less explored in RME‐ and DMLI‐based multivalent ECDs. Eh et al., evaluated the energy‐storage performance of a Cu–RME ECD device in water–DES hybrid electrolyte. With proper amount of reduced graphene oxide (rGO) coating on the FTO counter, the capacity of the ECD can be improved to ≈0.007 mAh cm^−2^.^[^ ^23^ ^]^

### 3.3 Thermal Management

Aside from smart window applications, multivalent ECDs capable of modulating NIR transmittance can also be adopted for thermal management applications. Dual‐band ECD with two PANI electrodes sandwiching a Zn frame can effectively regulate heat transfer. The temperature of a absorber plate under the PANI–ECD was measured to be 43.2, 31.8, and 27.2 °C when the ECD was switched to “bright,” “cool,” and “dark” modes, clearly demonstrating the ability of ECDs to block thermal transfer and their tunable energy‐saving capabilities under different modes.^[^ ^42^ ^]^ The electrodeposited reflective CuSn alloy film is resistant to surface oxidation and has significant NIR‐blocking property. As shown in Figure 7c, the surface temperature of electrodeposited CuSn/FTO (48 °C) is obviously lower than that of bare FTO (66.5 °C) when heated on a 180 °C hotplate, demonstrating the great potential of RME‐based ECDs for thermal management.^[^ ^23^ ^]^ The same group fabricated an black ECD based on RME of CuNi in gel electrolyte. Due to the excellent memory retention enabled by the gel electrolyte, the colored ECD is capable of blocking 19.5% IR radiation, showing excellent heat insulation property.^[^ ^100^ ^]^

### 3.4 Display

To achieve the requirements for display applications, multivalent‐ion‐based ECDs should manifest wide viewing angle, bistability, high contrast ratio, color tunability, and low power consumption.^[^ ^3^, ^11^ ^]^ RME‐based multivalent ECDs with color neutrality is less competitive for displays. Multivalent ECDs based on ion intercalation and DMLI with bistability and multicolor options are thus favored for display applications. Zn^2+^ intercalation into SVO can induce color variations from green, yellow to orange associated with the valance states of V, by sandwiching Zn frame in between two superimposed SVO electrodes, more available colors (orange, amber, yellow, brown, chartreuse, and green) can be obtained for multicolor displays.^[^ ^39^ ^]^ Alternatively, adopting similar device configuration, superimposing SVO with WO_3_ allows the assembly of a transmissive multicolor display. As shown in Figure 7d, the device can offer a wide range of colors by independently controlling the potentials of SVO and WO_3_ electrodes. A reflective multicolor display can also be obtained by superimposing SVO with W/WO_3_ electrodes.^[^ ^36^ ^]^ DMLI‐based multivalent ECDs exhibit high white light contrast ratios, wide viewing angle, and low power consumption. As shown in Figure 7e, based on the DMLI between Cu^+^/Cu^2+^ and M1, a non‐emissive transparent see‐through electrochromic display was assembled.^[^ ^11^ ^]^

## 4 Outlook and Perspectives

ECDs have found applications in dynamic windows,^[^ ^105^, ^106^, ^107^ ^]^ rear view mirrors,^[^ ^109^ ^]^ back cover of mobile phones,^[^ ^110^ ^]^ Ferrari glass roofs,^[^ ^111^ ^]^ etc. However, to the best of our knowledge, there are no commercially available multivalent‐ion‐based ECD products in the market, despite the research achievements in multivalent ECDs in recent years. There are still several issues to be addressed and more efforts should be devoted toward fabricating durable, reversible, scalable, and multifunctional multivalent ECDs.

### 4.1 Electrochromic Performance

There are several parameters to be considered when evaluating the electrochromic performance of multivalent ECDs, including Δ*T*, switching time, coloration efficiency, contrast ratio, cycling stability, color tunability, etc. Electrochromic performance comparison across different reports should be carefully handled as some of the performance parameters are intuitively contradictory, for example, larger Δ*T* requires longer switching time due to larger amount of charge consumption. To achieve optimal overall electrochromic performance in multivalent‐ion‐based ECDs, structure engineering of ECMs, formulation tuning of electrolytes, and configuration variation are necessary. However, it is still challenging to simultaneously achieve large Δ*T*, high coloration efficiency, fast switching, excellent cycling stability, and good bistability. Some performance parameters are more emphasized for certain applications. For example, large contrast ratio, fast switching, and excellent bistability are needed for display, large Δ*T*, and excellent cycling stability are favorable for smart window applications, while dual‐functional electrochromic‐energy‐storage multivalent ECDs need thicker working electrodes for more amount of charge storage. The design and construction of multivalent‐metal‐ion ECDs should be application oriented.

### 4.2 Electrochemical Mechanism

Similar to other electrochemical devices, there are also complicated physical/chemical variations within the multivalent‐metal‐ion ECDs during charge/discharge (coloration/bleaching). Advanced characterization techniques with respective advantages have been more than necessary to unveil the electrochemical mechanism in batteries and capacitors, etc.^[^ ^112^, ^113^ ^]^ The electrochemical mechanism in multivalent‐metal‐ion ECDs is comparatively less explored, and further efforts are to be devoted. Ion trapping in WO_3_ and TiO_2_ has been verified in Li^+^‐based ECDs, which accounts for the degraded cycling performance.^[^ ^114^ ^]^ Potentiostatic charging can de‐trap the ions and restore the reversibility. Similar phenomenon might also occur in multivalent‐metal‐ion‐intercalation/deintercalation‐based ECDs and requires further investigation. Aside from working electrodes, the counter electrodes in multivalent‐metal‐ion ECDs are less explored. In some cases, the employed metal counter electrodes (e.g., Zn or Al foil/mesh/frame) suffer from corrosion and side reactions in aqueous electrolytes with possible hydrogenation.^[^ ^31^ ^]^ Protection of metal counter electrodes are thus also necessary to ensure reversible and balanced charge/discharge in multivalent‐metal‐ion ECDs. Understanding possible redox reaction in electrolytes is also necessary.^[^ ^115^ ^]^

### 4.3 Practical Application

Lab‐scale performance optimization and electrochemical mechanism understanding are the prerequisites for commercial applications. However, more factors are to be considered for practical applications. Production of scalable multivalent ECDs requires efficient and high yield methods, for example, roll‐to‐roll coating.^[^ ^16^ ^]^ Device sealing should also be carefully handled to avoid electrolyte leakage, especially for large‐area ECDs. Antifreezing agents should be blended into the electrolytes of multivalent ECDs for outdoor applications, to avoid electrolyte volume expansion under low temperature. Larger‐area multivalent ECDs will bring problems of increased resistivity, nonuniform coloration (blooming effect) and slow switching kinetics.^[^ ^12^ ^]^ Homogeneous electric field distribution between working and counter electrodes should be well established, for example, by metal mesh/grids.^[^ ^41^, ^90^ ^]^ Cost analysis should also be completed for multivalent ECDs to penetrate into the market. For example, the cost of electrochromic smart windows should be kept lower than 500 USD m^−2^ and further reduced to 100 USD m^−2^.^[^ ^2^ ^]^

### 4.4 Multifunctional ECDs

Introduction of multifunctionality are key to broaden the future application of multivalent‐ion‐based ECDs. There have been several successful demonstrations of multifunctional multivalent ECDs, including energy‐storage flexible electrochromic battery and solar chargeable smart windows. For example, SDPANI was wrapped around a Zn wire, fabricating a fiber‐shaped electrochromic battery with a high volumetric capacity of 23.2 mAh cm^−3^.^[^ ^55^ ^]^ Such dual‐functional 1D electrochromic‐energy‐storage devices have great potential in smart wearable electronics. Similar to other electrochemical devices (supercapacitors,^[^ ^113^ ^]^ batteries,^[^ ^116^ ^]^ smart windows^[^ ^1^ ^]^), fabricating multifunctional multivalent‐ion‐based mechanically deformable (flexible and stretchable) ECDs, ECD‐sensors, ECD actuators, self‐chargeable (piezoelectric, triboelectric, thermoelectric) ECDs, etc., should be attempted, which will definitely bring more interests into multivalent ECDs.

There is still a long way to go before the merits of multivalent‐metal‐ion ECDs can be appreciated by the market. With thorough mechanism understanding, optimized electrochromic performance, efficient engineering and upscaling technology, cost effectiveness, and multifunctionality, the bottlenecks of multivalent‐metal‐ion ECDs will be solved, catalyzing the large‐scale production of fascinating multivalent electrochromic products in the foreseeable future.

## Conflict of Interest

The authors declare no conflict of interest.
